# Resist nanokirigami for multipurpose patterning

**DOI:** 10.1093/nsr/nwab231

**Published:** 2021-12-31

**Authors:** Qing Liu, Yiqin Chen, Zhanyong Feng, Zhiwen Shu, Huigao Duan

**Affiliations:** National Engineering Research Center for High Efficiency Grinding, State Key Laboratory of Advanced Design and Manufacturing for Vehicle Body, College of Mechanical and Vehicle Engineering, Hunan University, Changsha410082, China; National Engineering Research Center for High Efficiency Grinding, State Key Laboratory of Advanced Design and Manufacturing for Vehicle Body, College of Mechanical and Vehicle Engineering, Hunan University, Changsha410082, China; National Engineering Research Center for High Efficiency Grinding, State Key Laboratory of Advanced Design and Manufacturing for Vehicle Body, College of Mechanical and Vehicle Engineering, Hunan University, Changsha410082, China; National Engineering Research Center for High Efficiency Grinding, State Key Laboratory of Advanced Design and Manufacturing for Vehicle Body, College of Mechanical and Vehicle Engineering, Hunan University, Changsha410082, China; National Engineering Research Center for High Efficiency Grinding, State Key Laboratory of Advanced Design and Manufacturing for Vehicle Body, College of Mechanical and Vehicle Engineering, Hunan University, Changsha410082, China

**Keywords:** nanokirigami, multi-scale patterning, multipurpose patterning, electron-beam lithography, inverse structure

## Abstract

Resist-based patterning solutions play essential roles in modern micro- and nanoscale science and technology. The commonly used ‘resist’ patterning strategy depends on selective-area scission or cross-linking of resist molecules under the action of an energy beam. In this work, we propose and demonstrate a different resist-patterning strategy, termed ‘resist nanokirigami’, in which the resist structures are defined by their outlines and revealed by selective mechanical peeling of the unwanted resist film. Unlike conventional resist-based patterning processes, the final resist-nanokirigami structures do not undergo exposure and the exposure area is dramatically reduced. With these two advantages, a variety of functional structures that are difficult or impossible to fabricate by conventional processes, such as inverse nanostructures and their oligomers, multi-scale electrodes and freestanding plasmonic nanogaps, can be easily achieved with much higher efficiency. Thus, with its unique and complementary capabilities, the resist-nanokirigami process provides a new patterning solution that expands the family of lithography techniques and will play a significant role in fabricating multi-scale functional structures.

## Teaser

This paper demonstrates a novel lithography strategy based on resist nanokirigami, which can greatly improve the processing efficiency and reduce the proximity effect of electron beam lithography to define multiscale resist structures for multipurpose applications.

## INTRODUCTION

Advanced micro- and nanoscale patterning techniques are essential in modern micro- and nanoscale science and technology. Among the various techniques available, resist-based lithographic methods, mainly including photolithography and maskless direct writing, are the most sophisticated and important [1–7]. Conventional resist-patterning processes depend on selective-area scission or cross-linking of resist molecules under exposure to an energy beam. Upon removing the unwanted parts of the resist film in a developer solution, the final resist pattern is obtained. The resist is defined as either a positive- or negative-tone resist according to whether the exposed sections are removed or remain [8,9]. In combination with film-deposition and pattern-transfer (i.e. etch and lift-off) processes [10–12], different kinds of functional structures and devices can be fabricated based on a predefined positive- or negative-tone resist pattern.

Although the resist-based patterning process has been standardized for several decades since its first use, it still presents problems when used to define certain functional structures. For example, in high-resolution direct-writing techniques, usually referred to as focused electron- or ion-beam lithography, the standard resist-based patterning process requires point-by-point exposure of the target resist structures, leading to extremely low throughput [13] and an unavoidable proximity effect when defining multi-scale patterns [14–16]. Another problem is the negative-tone-resist-based lift-off process required for inverse functional structures [17,18]. The lift-off process is easily achieved with positive-tone resists because they are soluble in many solvents. However, the negative-tone-resist-based lift-off process is challenging because the predefined negative-tone resists have to be sufficiently cross linked in the standard lithographic process. Such cross-linked resist molecules are difficult to remove owing to the formation of networked chains with very high molecular weights, thus limiting its applicability to numerous functional structures and devices [19]. Along with the increased requirements of resist patterning exotic structures with complicated features for emerging applications, developing advanced-lithography solutions with improved capabilities has always been highly demanded.

In this work, we propose and demonstrate a different lithography strategy that complements the capabilities of existing standard lithographic processes. This new strategy, termed ‘resist nanokirigami’, is achieved by combining nanoscale direct writing and selective mechanical peeling. Compared with existing standard lithographic processes, the nanokirigami strategy only requires the exposure of the outlines of the target resist patterns and the resist film outside the outlines is removed by mechanical peeling. This strategy allows much smaller exposure areas for multi-scale resist patterns, enabling significantly enhanced patterning efficiency and mitigating proximity effects. More importantly, the remaining resist patterns and peeled resist film do not undergo exposure and they can be functionalized as either negative-tone or positive-tone templates for multipurpose applications. A series of functional structures and device applications that are difficult or impossible to achieve with existing lithography solutions are explored to demonstrate the uniqueness and robustness of the resist-nanokirigami strategy.

## RESULTS AND DISCUSSION

**Figure 1.**
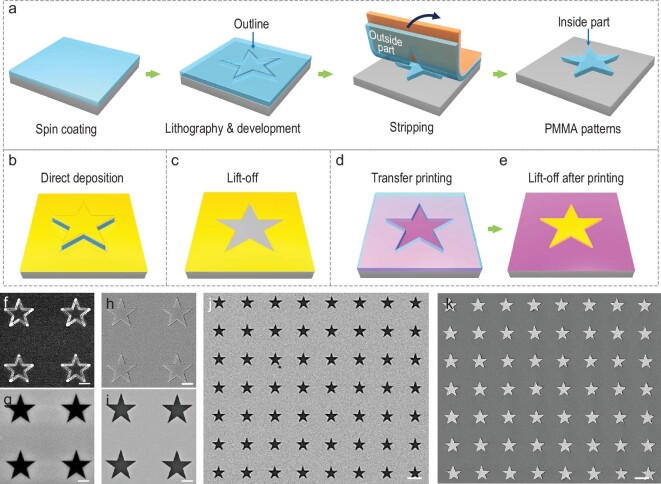
Schematic and demonstration of resist nanokirigami for multipurpose applications. (a) Fabrication process flow of the resist-nanokirigami strategy. (b)–(e) Schematics of different applications, including (b) direct deposition on inside-resist structures, (c) lift-off for inverse structures, (d) transfer printing of the outside-resist film and (e) lift-off based on the transferred outside-resist film. (f)–(i) SEM images showing different experimental steps, including (f) formation of outlines, (g) after selective peeling of the outside-resist film, (h) after deposition of a functional film and (i) after lift-off. (j) and (k) SEM images showing a large array of the final functional structures based on the remaining inside-resist template and the transferred outside-resist film, respectively. Scale bars: 500 nm in (f)–(i); 2 μm in (j) and (k).

The basic concept and a typical experimental demonstration of our resist-nanokirigami strategy for multipurpose patterning are illustrated in Fig. 1. Figure 1a)shows the process flow of the resist-nanokirigami process. In the first step (i), a positive-tone resist such as polymethyl methacrylate (PMMA) is spin-coated onto a pre-decorated substrate. The substrate is pre-decorated with an anti-adhesion self-assembled monolayer material such as hexamethyldisilazane (HMDS) to guarantee the successful peeling of the resist from the substrate in the final mechanical peeling step [20]. In the following step (ii), electron-beam lithography (EBL) is conducted to define the outlines of the target structures. The outlines split the resist film into inside and outside parts. By peeling the resist film with a conformally adhered tape (iii), the outside resist is selectively peeled off, while the inside-resist structures remain on the substrate (iv). The detailed experimental process is shown in Fig. S1.

Compared with conventional resist-based lithographic processes, the resist-nanokirigami process has several unique advantages, as illustrated in Fig. S2. First, the areal structures are defined by their single-pixel outlines. Therefore, the exposure area is drastically reduced and thus the patterning efficiency is significantly enhanced, especially for these large structures, as calculated in Fig. S3. For example, the exposure area is reduced by a factor of 10 000 when defining a structure with a diameter of 800 μm. Second, with a much smaller exposure area, the proximity effect caused by electron scattering is largely mitigated, enabling improved patterning accuracy for defining multi-scale structures with sharp corners or small gaps. Third, the remaining inside-resist structure, acting as a negative-tone structure, does not undergo any exposure, so it can be used as a sacrificial template for wet lift-off applications. This advantage is extremely useful in defining inverse functional structures, which is difficult to realize with common negative-tone resist processes owing to their cross-linking-induced insolubility [19,21]. Finally, the outside part of the resist on the peeling tape is also usable. For example, it can be transferred to another substrate and act as a positive-tone structure for applications, such as for the processing of sensitive materials [22,23], non-planar substrates and flexible devices [24].

The above advantages make the resist-nanokirigami process suitable for multiple applications, as shown in Fig. 1b–e. For instance, the remaining inside structure can be used as a template for the direct deposition of functional films (1b) and for subsequent lift-off (1c). Note that one unique advantage of positive-tone resists (e.g. PMMA) is that multi-layer resists with different kinds of sensitivity can be used for realizing undercut or freestanding structures, making directly deposited functional structures more useful and the lift-off process easier [25,26]. Conversely, the outside resist on the adhesion tape can be transferred onto an appropriate substrate (1d) via transfer printing. The transferred resist can then be used as a template to define functional structures via the appropriate combination of film-deposition and lift-off processes.

As a demonstration, Fig. 1f–i shows the experimental results of the nanokirigami concept. In Fig. 1f, PMMA star structures are first defined by their nanoscale outlines, separating them from the PMMA film. Upon selective mechanical peeling, the outside PMMA film is removed and only the PMMA star structures remain (Fig. 1g). Figure 1h)and i depict the structures after film deposition and lift-off, corresponding to the schematics in Fig. 1b)and c, respectively. The whole process is robust and a large array of inverse metallic star structures based on the inside-resist template after lift-off is shown in Fig. 1j. Figure 1k)shows the metallic star structures defined by the transfer-printed outside-resist film after lift-off, demonstrating the feasibility of the application illustrated in Fig. 1d)and e. Note that the above resist processes can be applied to various deposition methods and materials, making them general and versatile for different kinds of applications compared to previously developed sketch-and-peel lithography [27,28] that is only applicable for evaporated metallic structures.

**Figure 2.**
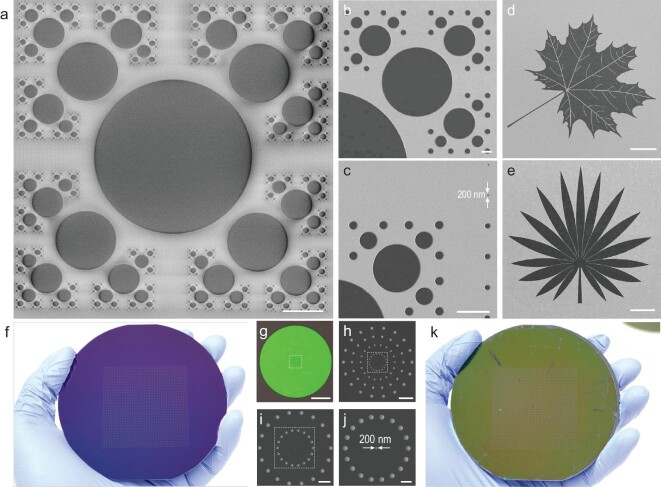
Multi-scale patterning with the resist-nanokirigami process. (a) SEM image of a fractal pattern consisting of disks with the diameters from 200 nm to 200 μm. (b) and (c) Enlarged SEM images of selected areas in (a). (d) and (e) SEM images showing two kinds of multi-scale resist structures. (f) Photograph showing the feasibility of 4-inch-wafer-scale resist nanokirigami. (g) Photograph of a unit cell of the sample shown in (f). (h)–(j) SEM images showing micro- and nanoholes in the unit cell. (k) Photograph showing the transfer-printed resist film peeled from the sample shown in (f). Scale bars: 50 μm in (a), (d), (e) and (g); 5 μm in (b), (c) and (h); 2 μm in (i); 1 μm in (j).

To further demonstrate the robustness of the resist-nanokirigami process, multi-scale resist patterns with different feature sizes (from several hundreds of nanometers to several hundreds of micrometers) were fabricated. Figure 2a)shows a fabricated fractal pattern consisting of multi-scale disks. The middle disk has a diameter of 200 μm and the smallest disk has a diameter of 200 nm. Upon carefully checking the whole pattern, it was determined that all the desired disks were obtained, demonstrating a 100% yield for disks with different scales. Figure 2b)and c are magnified scanning electron microscopy (SEM) images of two selected corners in Fig. 2a, showing that the structures are well defined with smooth contours. Further systematic statistical experiments indicated that the resist-nanokirigami process is applicable to structures with sizes up to the millimeter scale and down to 35 nm, as illustrated by Fig. S4. It should be noted that this process does not present an improvement in terms of throughput for ultra-small nanoscale structures owing to the exposure areas for the two strategies being almost equivalent at this scale. In addition, because this process relies on interfacial fracture propagation, it may be challenging to define densely packed structures when the space between the structures is too small (e.g. 100 nm, depending on the resist thickness). For this case, overall exposure of the space area is required, as illustrated in Fig. S5.

Nevertheless, with its generally enhanced throughput and mitigated proximity effect, the resist-nanokirigami process is extremely useful for rapid patterning of multi-scale structures. As seen in Fig. 2d)and e, two kinds of leaves that have both macroscale and nanoscale features were defined. For these multi-scale structures with sizes up to several hundreds of micrometers (e.g. diameter = 200 μm), the actual exposure area can be reduced by a factor of 2500 considering that only the outline, which has a linewidth less than 20 nm, is exposed. With this advantage, wafer-scale fabrication of multi-scale structures with a laboratory EBL machine becomes possible, as shown in Fig. 2f. The unit cell for the sample shown in Fig. 2f)is a 200-μm microdisk with embedded microholes and nanoholes. The details of the structures in the unit cell are shown in Fig. 2g–j. The realization of both hole and disk structures demonstrates that both positive- and negative-tone structures can be achieved using resist nanokirigami. Figure 2k)shows the transfer-printed PMMA outside-resist film from the tape, confirming the possibility of reusing peeled resists at the wafer scale.

**Figure 3.**
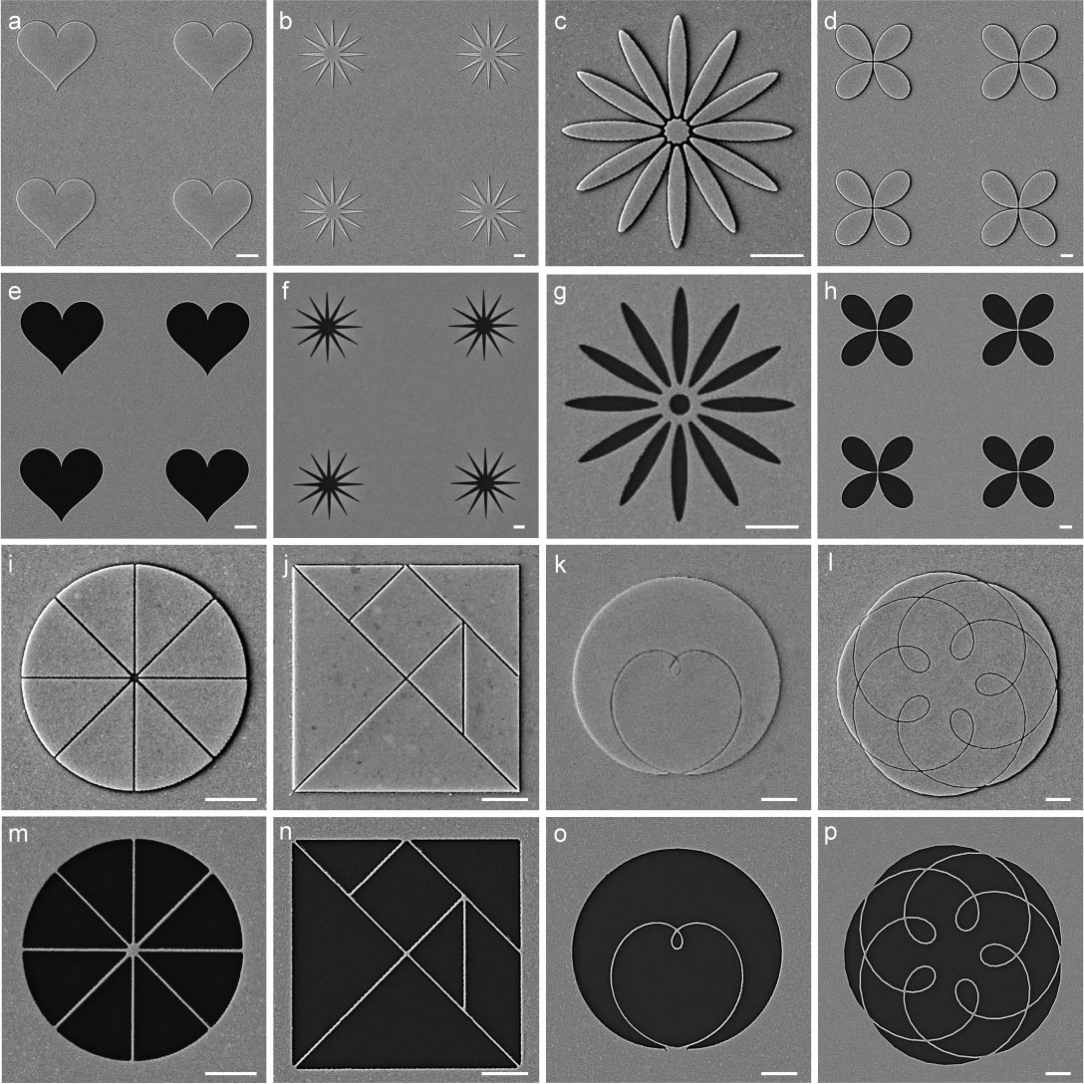
Fabrication of complex metallic structures with resist nanokirigami. (a)–(c) Structures containing sharp features. (d) A flower-like structure formed by four closely packed leaves. (e)–(h) The corresponding inverse metallic structures after lift-off. (i)–(l) Multi-scale structures divided by nanolines. (m)–(p) The corresponding inverse metallic structures after lift-off. All scale bars: 1 μm.

The nanokirigami process provides easy realization of multi-scale and inverse functional patterns that are difficult to achieve with conventional resist-based lithography processes. A series of typical examples are provided in Fig. 3. Figure 3a–c show three different patterns with either fine features or sharp corners, in which 30-nm-thick gold films were deposited. After lift-off, the inverse gold patterns are obtained, as seen in Fig. 3e–g, respectively. For patterns that need to be densely packed, e.g. oligomers, shared boundaries can be employed to realize the smallest possible features. Fig. 3d)shows a flower-like pattern consisting of four leaves, in which they are separated by exposed single-pixel lines. After metallic film deposition, metallic nanogaps can be achieved. Consequently, four inverse metallic leaves separated by fine metallic lines can be obtained after lift-off. Note again that reliable lift-off is enabled by the solubility of the positive resist and the bilayer resist process, which is an intrinsic advantage of positive resists in this process.

With a shared boundary design, a pattern can be split into multiple elements by arbitrary lines. Figure 3i–l show four typical patterns that are divided either by straight lines (3i, 3j) or by mathematical curves (3k, 3l), and their corresponding metallic structures after lift-off are shown in Fig. 3m–p. The structures obtained have smooth edges and the nanolines have a linewidth of ∼30 nm. Such inverse metallic structures are impossible to fabricate with conventional processes, confirming the unique applicability and utility of the resist-nanokirigami strategy. Note that all the processes discussed above can be realized for large arrays and more complicated patterns can also be achieved, as shown in Figs S6–S8.

**Figure 4.**
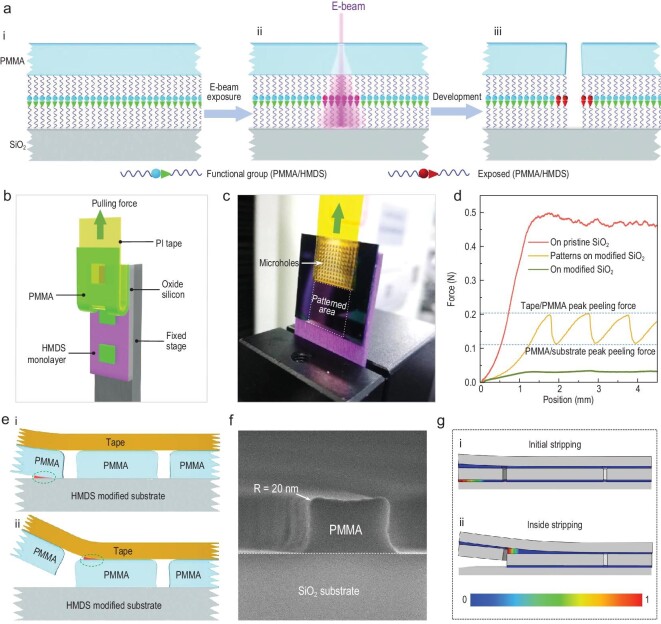
Analysis of the selective-peeling mechanics in resist nanokirigami. (a) Schematic illustration of adhesion transition enabled by high-energy electron irradiation. (b) 3D schematic model showing the peeling-test set-up and the peeling process. (c) Digital photograph showing a patterned PMMA sample on an HMDS-modified substrate during stripping. (d) Force–displacement curves on three different substrates: (red) pristine SiO_2_ without HMDS modification and patterns; (orange) HMDS-modified SiO_2_ substrate with EBL-defined PMMA patterns; and (green) HMDS-modified SiO_2_ substrate without patterns. (e) Schematic illustration of the stripping scenarios of the outside film (i) and inside structure (ii). (f) Cross-sectional SEM image showing the geometrical profile of the PMMA structure remaining on the substrate after peeling, demonstrating obvious fillets (*R* = 20 nm) at the two top corners. (g) Interfacial damage distribution of the sample at the moment of initial stripping of the outside film and (i) the inside structure (ii). Zero interfacial adhesion was set at the initial region of the sample to mimic the pre-cracks from the defects at the edge of the sample. Color bar represents the normalized level of interface damage, where 0 indicates no damage and 1 indicates complete separation.

The mechanism of the resist-nanokirigami process is illustrated in Fig. 4. The key question is how the outside-resist film is selectively stripped while the inside-resist structures can well remain during the tape peeling. Our hypothesized answer is as follows. First, the anti-adhesion molecules at the resist–substrate interface (i.e. HMDS) facilitate the peeling of the resist, but they can be modified by electron irradiation, leading to the loss of their anti-adhesion property. Second, the inside-resist structures are mechanically more stable during the peeling due to the absence of pre-cracks.

Figure 4a)shows the schematics of electron irradiation of HMDS molecules. In the initial step, a PMMA-resist film is spin-coated onto an HMDS-modified SiO_2_ substrate (Step i). Note that HMDS is commonly considered as an adhesion promoter, but the mechanical adhesion between the resist and the substrate is in fact significantly weakened after HMDS modification, as reported by some literature [29] and verified by our experiments shown below. In detail, since HMDS leaves methyl groups on the surface of the substrate, only Van der Waals forces are allowed to interact with PMMA, thus exhibiting weak mechanical adhesion [30]. Subsequently, it is processed with the contour exposure using a focused electron beam (Step ii). The high-energy electron irradiation results in two effects. The PMMA resist in this area is exposed. However, the underlying HMDS layer on the substrate is also damaged (indicated by the red box), and the modified HMDS layer becomes more highly adhered. Finally, the exposed PMMA is removed in the development process. The inevitable occurrence of electron scattering during EBL leads to the lateral extension of deposited energy and damages the HMDS layer underneath the PMMA resist on both sides of the contour (Step iii). Therefore, two parallel annular high-adhesion regions are formed at the edge of the inside and outside of the structure, respectively. The electron-irradiation-induced modification of HMDS has been reported by previous literature [31–33] and the enhanced adhesion after irradiation is also demonstrated by several control experiments in this, as illustrated in Fig. S9.

To verify the importance of HMDS in this process, the adhesion force between the resist and different substrates was measured via mechanical peeling tests [34]. Three different kinds of samples were used for measurements, i.e. a PMMA-resist film on pristine SiO_2_ substrate without an HMDS monolayer, a PMMA-resist film on an HMDS-modified SiO_2_ substrate and a PMMA-resist film with EBL-defined contour patterns on an HMDS-modified SiO_2_ substrate. Figure 4b)schematizes the peeling process for a patterned PMMA film on an HMDS-modified SiO_2_ substrate and the corresponding experimental scenario is shown in Fig. 4c. Figure 4d)shows the measurement results. The PMMA-resist film on HMDS-modified substrate can be easily peeled off with little force (0.03 N, green curve), indicating its weak adhesion. In contrast, the PMMA-resist film exhibits extremely strong adhesion to the pristine substrate without an HMDS monolayer and cannot be stripped from the substrate, causing the direct stripping of the peeling tape. Therefore, the measured peeling force (0.45 N, red curve) actually reflects the adhesion between the peeling tape and the PMMA resist, while the adhesion between the PMMA resist and the substrate is even stronger, implying that the adhesion force of the unmodified surface is at least 15× that for the HMDS-modified surface.

For the patterned sample, the measured peeling force (orange curve) initially rises to a higher value and rapidly decreases to a low point (labeled with a dashed blue line). Due to the periodicity of the predefined patterns, the measured force curve as a function of displacement presents periodic oscillation behavior. By correlating the peeling-force curve with the pattern positions, the high point in this curve is ascribed to the occurrence of the stripping at the interface between the PMMA and the tape, and the low point indicates the peeling at the interface between the PMMA and the HMDS-modified substrate. After peeling, the inside PMMA structure array remains on the substrate (see in Fig. S10).

From the peeling-force curves, we can intuitively conclude that resist nanokirigami is achieved via two factors, i.e. the extremely weak adhesion of the outside-resist film and the higher mechanical stability of the inside-resist pattern. Reliable experimental results can also be achieved using other peeling tapes, such as thermal release tape. The corresponding adhesion analysis and reliability statistics are shown in Fig. S11.

It is well known that interfacial pre-cracking benefits film stripping from a flat surface because the pre-cracking results in stress concentration at the front of the fracture. For peeling the outside film off, the nanoscale defects at the edge of the sample serve as pre-cracks at the initial stripping. When undergoing a peeling force, the pre-cracks become an obvious interfacial fracture and then result in stress concentration at the front of fracture, as marked by the red area in Fig. 4e–i. When the peeling force continues, the interfacial fracture between the PMMA resist and the substrate propagates. When the fracture propagates to the EBL-defined contours, the fracture at the patterned area extends to the interface between the tape and PMMA, as shown in Fig. 4e–ii. Simultaneously, the fracture at the outside of the patterns continues to propagate at the interface between the PMMA and the substrate, leading to the removal of all the PMMA outside-resist film.

The higher mechanical stability of the inside PMMA pattern is understandable because it does not undergo pre-cracking. Instead, the pre-cracks are more likely to exist at the intersection of the top contour of the PMMA pattern with the peeling tape, as marked by the red arrow in Fig. 4e–ii. More details of crack propagation at the PMMA–HMDS and tape–PMMA interfaces are shown in Fig. S12. Figure 4f)shows a cross-sectional SEM image of a typical EBL-defined PMMA structure, highlighting the round profile at the edge of the structure. The round profile is thought to benefit the formation of pre-cracks between the peeling tape and the PMMA structure, facilitating the stripping of the tape from the PMMA structure.

To further investigate the origin of the mechanical stability of the inside PMMA patterns, the interfacial damage behavior in the two stripping scenarios for the outside-resist film and the inside structure was analysed using the finite-element method. A cohesiveness analysis method was used and the traction–separation (*T–δ*) law of the cohesive-zone model is shown in Fig. S13. Two main considerations are included in the simulation model. First, the round profile of the PMMA structure based on the SEM image was used. Second, stronger adhesion was used at the edge of the interface between the inside PMMA structure and the substrate to account for the damage to HMDS molecules. The size of the inside pattern was set to 5 μm and the adhesion enhancement area was set to 40 nm. The simulation results are given in Fig. 4g. The damage mappings indicate that large interfacial damage tends to appear around pre-crack sites. As shown in Fig. 4g–i, for initial stripping, defects at the PMMA–substrate interface serve as pre-cracks that benefit the formation of fractures at the edge of the sample. For stripping inside the PMMA structure, the maximal damage appears at the round edge of the tape–PMMA interface, indicating the formation of fractures, as shown by Fig. 4g–ii. A more detailed simulation and analysis is shown in Fig. S14. Further systematic simulations verify the synergetic role of the local adhesion enhancement of the contour PMMA resist on the stability of the inner structures, as shown in Fig. S15. Note that our simulations only provide a qualitative consideration of the possible factors influencing the selective-peeling phenomenon. The quantitative mechanics are much more complicated considering the actual material properties and peeling parameters, and they should be further investigated in future work.

**Figure 5.**
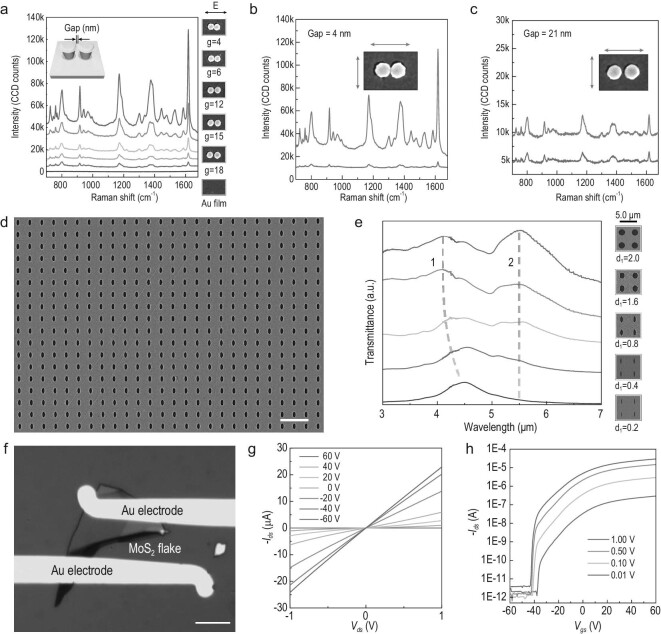
Applications of three typical structures fabricated using the nanokirigami process. (a) Raman spectra of freestanding gold nanogaps on nanodisk dimers with different gap sizes. (b) and (c) Polarization-dependent SERS of gold nanodisk dimers with 4-nm (b) and 21-nm gaps (c) under linear excitation parallel and perpendicular to the dimer axis. (d) SEM image of an extraordinary optical transmission (EOT) infrared filter consisting of periodic elliptical microholes. (e) Spectral responses of EOT devices with different curvature radii and pitches. The left column of the SEM images represents decreasing *d_1_* values (the length of the *x*-axis in the elliptical hole) from bottom to top. The right column of the SEM images represents increasing pitch in the elliptical hole array. (f) Photograph of an MoS_2_ transistor fabricated by the transferred outside resist. (g) Output *I_ds_*–*V_ds_* curves of an MoS_2_ transistor under different gate voltages from –60 to 60 V. (h) ON/OFF ratio curves with different source–drain bias voltages. Scale bars: 10 μm in (d) and (f).

The unique advantages of this nanokirigami process can provide exclusive solutions for various device applications in nano optics, plasmonics and electronics, as illustrated in Fig. 1. In the following section, we demonstrate these applications experimentally and the results are shown in Fig. 5.

Corresponding to the scenario in Fig. 1b, the unique negative-tone-like bilayer resist structures enabled by the nanokirigami process can be used to reliably define freestanding ultra-small plasmonic nanogaps for chemical sensing and surface-enhanced spectroscopy [35–38]. A detailed fabrication flow is shown in Fig. S16. Freestanding structures are thought to significantly benefit plasmonic performance. As shown in the schematic inset of Fig. 5a, a plasmonic nanogap can be fabricated by direct metal deposition on two closely defined PMMA nanoposts with nanoscale separation. Owing to the lateral growth that occurs during deposition, the resultant metallic gap size is smaller than that of the two original resist nanoposts before deposition. An average reduction in gap size of 30 nm after metal deposition was observed for a fixed metal thickness of 30 nm, as indicated by the statistical results given in Figs S17 and S18. Note that the freestanding nature of the template nanoposts is a key requirement to implement this fabrication process and avoid sidewall deposition. Furthermore, the initial small gap defined by single-pixel-line exposure in the resist-nanokirigami strategy is also important for achieving ultra-small gaps. Figure 5a)shows the surface-enhanced Raman spectroscopy (SERS) performance of a gold nanodisk dimer with differently sized freestanding gaps. The intensity of fingerprint spectral peaks from the analyser (crystal violet) drastically increases with the reduction in gap size. The enhancement factor measured on a single dimer with a 4-nm gap is higher than that enabled by a single particle with an 18-nm gap size by a factor of >25. In Fig. 5b, for the dimer with a 4-nm gap, the SERS signal is more than 10× stronger when the linear excitation is parallel (orange line) to the axis of the dimer compared with that from perpendicular polarization (green line). In contrast, for the dimer with a 21-nm gap, the SERS signal of parallel excitation is only 2× stronger than the perpendicular one, as seen in Fig. 5c. These experimental results agree well with the simulations and the corresponding electric field-distribution analysis is shown in Fig. S19. The above results indicate that the nanokirigami process provides a more feasible approach to reliably fabricating single-digit-nanometer gaps for plasmonic applications.

Figure 5d)shows an SEM image of a silver microhole array for transmission applications [39] that corresponds to the fabrication concept proposed in Fig. 1c. More details of the process and Ag microhole arrays are shown in Figs S20 and S21, respectively. Such microhole arrays are difficult to achieve owing to difficulties associated with silver dry etching and negative-tone-resist-based lift-off. With the resist-nanokirigami process, a metallic microhole array can be defined with enhanced throughput, geometry precision and edge smoothness. By varying the ovality of silver microholes, their transmission spectra can be finely tuned [40], as seen in Fig. 5e. Two main peaks (marked as ‘1’ and ‘2’) were obtained. When the length (*d_1_*) of the ellipsoid in the short axis increases with a constant 2-μm long axis (see in SEM images in the right column), Peak 1 presents a smooth blue shift from 4.5 to 4.1 μm and Peak 2 is fixed at 5.5 μm. The measured spectra agree well with the simulated spectra in terms of the resonant modes and the shift trends, as indicated in Fig. S22. Further control experiments and simulations suggested that the resonant mode of Peak 1 is a localized surface plasmon along the short-axis polarization and Peak 2 is lattice mode caused by a (1, 0)-propagating surface plasmon at the gold–CaF_2_ interface. The blue shift of Peak 1 is due to fading capacitive coupling of localized surface plasmon dipolar polaritons in holes with increasing *d_1_*. More details of the fabrication process, spectra measurements and spectral analysis are provided in the supplementary experimental section, Figs S23 and S24.

As proposed in Fig. 1d)and e, the stripped outside film can serve as a transferrable stencil mask for lift-off applications. Together with its rapid outline patterning, the nanokirigami process is particularly suitable for defining electrode pads for nanoelectronic devices. Furthermore, after metal deposition, the printed resist film can be further removed by dry stripping for pattern transfer. Such all-dry-transfer processes avoid electron irradiation damage and wet-solution-induced contamination on vulnerable 2D materials [41–43] such as MoS_2_. As a demonstration, Fig. 5f)shows a nanoelectronic device based on a MoS_2_ flake, in which the electrode pads are defined via the transferred outside-resist film. The detailed fabrication process flow is given by Fig. S25. In Fig. 5g, the linear *I*_ds_ vs. *V*_ds_ output curve shows gold–MoS_2_ junctions is ohmic contact. Fig. 5h)shows that the *I*_ds_–*V*_gs_ transfer curve and the ON/OFF ratio can achieve a value as high as ∼10^7^, which proves that the high performance of 2D material transistors is enabled by our all-dry process.

## CONCLUSION

We have developed a new resist-based patterning strategy by combining nanoscale direct-writing and mechanical selective-peeling processes. Termed ‘resist nanokirigami’, this new strategy is enabled by engineering the adhesion of the resist with the substrate and the unique interfacial fracture mechanics during the peeling process. Compared with conventional standard lithographic processes, resist nanokirigami presents exclusive advantages for defining multi-scale structures with significantly enhanced efficiency and precision as well as the ability to transform positive-tone resists to negative-tone resists for multiple applications. Several kinds of functional structures and devices that are difficult to fabricate with existing lithographic processes have been shown as a means to demonstrate the uniqueness and robustness of this nanokirigami strategy. With its unique capabilities, this strategy provides an extra resist-based patterning solution that complements current lithography techniques and constitutes a new member of the advanced-lithography family of solutions. Our work provides an example using interfacial fracture mechanics to expand the capabilities of standard lithographic processes, paving the way for new research directions in mechanics-enabled micro- and nanoscale lithography that is also applicable to other resist-based lithographic techniques, such as photolithography, tip-based lithography and focused-ion-beam lithography. By further standardizing the mechanical processes via specifically designed mechanical processing tools, processing materials and optimized processing parameters after completely understanding the underlying mechanics in the processes, we believe that this strategy will play an important and irreplaceable role in fabricating functional structures and devices for practical applications.

## METHODS

### EBL

To enable the PMMA layer to be peeled off from substrates, we first used hexamethyldisilazane (HMDS) to modify an oxidized silicon substrate with a self-assembled monolayer in a sealed chamber at 120°C for 30 min. Following surface modification, a bilayer PMMA (950 k (top)/200 k (bottom)) resist was spin-coated onto the modified SiO_2_ substrate and prebaked on a hotplate at 180°C for 5 min after each spin-coating step. To make the process more stable, the PMMA resist should be sufficiently thick. We found that a resist thinner than 50 nm may lead to decrease yield due to its reduced mechanical strength to support the interfacial fracture propagation. Subsequently, EBL was performed using a Raith 150^TWO^ electron-beam direct-writing system with an accelerating voltage of 30 kV and a beam current of 180 pA. The write field was set to 100 × 100 μm and the step size was 2 nm. A single-pixel-line geometry element was used for layout designs. After finishing the exposure, the sample was developed in the methyl isobutyl ketone and isopropanol (IPA) solution mixture (vol ratio = 1 : 3) at –18°C for 1 min and immediately immersed in IPA solution for 1 min to quench development. Finally, the sample was blow-dried under a steady stream of nitrogen gas.

### Peeling off and transfer printing

In most experiments, polyimide (PI) tape (QDL-GW-008) was used for peeling because the outside PMMA film needs to be removed. First, the PI tape was tightly pasted onto the patterned PMMA film. Subsequently, the pasted PI tape was peeled off at a rate of 10–30 mm/min. For some specific applications, the stripped outside PMMA film as a stencil template needed to be printed on a functional material or substrate, so we needed to release the PMMA stencil for use on other materials. Smart tapes that change their adhesiveness under external stimuli can be converted to low-adhesion forms; for example, peeling-rate-dependent adhesive polydimethylsiloxane (PDMS) or thermal release tape. When peeling using PDMS, we conducted the peeling off at a rate of >100 mm/s to strip the outside of the PMMA stencil and with a rate of <1 mm/s to retrieve the PDMS after PMMA printing. In the thermal-release-tape-assisted transfer printing of PMMA stencils, pasting tape and peeling-off was performed by grabbing the outside PMMA film at room temperature, and then the sample was heated to 90°C after printing the PMMA stencil to perform complete retrieval of the tape from the PMMA surface. Note that an appropriate resist thickness, tape adhesion and transfer process is required to decrease the possibility of buckling, corrugation or cracking.

## Supplementary Material

nwab231_Supplemental_File
