# Supplementary material for: Resist nanokirigami for multipurpose patterning
Source: Natl Sci Rev. 2021 Dec 31;9(11):nwab231. doi: 10.1093/nsr/nwab231 (PMC9746683; doi:10.1093/nsr/nwab231)
Supplement: nwab231_Supplemental_File [file nwab231_supplemental_file.pdf]

Supplementary Information for

# Resist Nanokirigami for Multipurpose Patterning

Qing Liu#, Yiqin Chen#, Zhanyong Feng, Zhiwen Shu, Huigao Duan*

National Engineering Research Center for High Efficiency Grinding, State Key Laboratory of Advanced Design and Manufacturing for Vehicle Body, College of Mechanical and Vehicle Engineering, Hunan University, Changsha, 410082, China

*Corresponding author. E-mail: duanhg@hnu.edu.cn

## Experimental Section:

### 1. Peeling Test

All peeling tests were carried out on a stretching test equipment. PI tape with a width of 10 mm was pasted on PMMA surface. One head connect to the PI tape, and tested samples were fixed on the other head. The peeling force was measured by a digital force gauge (ZQ-990L). The peeling-off test was executed with the reverse stripping (180°) of PI tape.

### 2. FEM Simulation

The mechanical analysis was used by the commercial finite element analysis software (ABAQUS 2019). A quasi-static model was established to simulate the peeling-off process. To improve the computational efficiency, 3D symmetric modeling with respect to the xz-plane was set in simulation domain. Reduced iteration step size and fine mesh and were used to improve the accuracy in simulation. Considering the geometry of PMMA nanodisk, it was set as a solid unit in analysis modeling, while silicon substrate was set as discrete rigid. The cohesiveness analysis method was used to set the damage at the connection part and PMMA/substrate interface[[1]], and the constitutive behavior of the interface was assumed as a bi-linear Traction-Separation law composed of an elastic stage and a softening stage (Figure S10). The elastic modulus (E) and Poisson's ratio ($v$) are $E_{PMMA}$ = 4 GPa, $v_{PMMA}$ = 0.33[[2]]. The corresponding parameters (*e.g.*, interface strength/$\sigma$ and interface energy/$W$) were set as follows: $W_{PI/PMMA} = 1 \times 10^{-4}$ N/mm, $\sigma_{PI/PMMA,\ n}$ =1 MPa; $W_{PMMA/substrate} = 4 \times 10^{-6}$ N/mm, $\sigma_{PMMA/substrate,\ n}$=0.5 MPa; $W_{PMMA/substrate\ (steady\ area)} = 5 \times 10^{-4}$ N/mm, $\sigma_{PMMA/substrate\ (steady\ area),\ n}$ = 8 MPa.

### 3. FDTD Simulation

The finite-difference time-domain (FDTD) simulations (Lumerical Solutions Inc., Version 8.15) were performed to analyze the EOT spectral response and field

distribution profiles. The boundary conditions were set to be periodic in X- and Y-axes and perfectly matched layer (PML) along the propagation of electromagnetic waves (Z-axis). The dimensions of 3D model in geometry were referred to that measured in SEM images of Figure S17. A fine mesh with 1 nm in X, Y coordinates and 2 nm in Z coordinate was added to encase the 3D model of nanodisk dimer in simulation domain. The dispersion of gold was from Johnson-Christy database. The refractive index of quartz, $CaF_2$ and PMMA were set to be the constant of 1.48, 1.39 and 1.46, respectively.

## 4. Morphology Characterization

The morphology of defined structures was characterized on a field-emission scanning electron microscope (FE-SEM, SIGMA-HD, Carl-Zeiss) at an accelerating voltage of 5 kV with the working distance of 6 mm. The optical characterization was performed on a Carl-Zeiss microscope (AXIO-10) equipped with 5× (0.13 N.A.), 10× (0.25 N.A.), 20× (0.4 N.A.), 50× (0.75 N.A.), and 100× (0.85 N.A.) objectives.

## 5. Raman Measurement

The periodic100-nm-diameter gold nanodisk dimers with freestanding gaps on $SiO_2$/Si substrate were immersed in the solution of crystal violet dissolved in ethanol with a concentration of $10^{-5}$ M for 15 hours and finally blow-dried by a steady nitrogen gas stream. A confocal Raman microscope (Renishaw inVia) was used to evaluate the SERS performance of varied-gap-size Ag nanodisk dimers. The excitation wavelength is 633 nm from He-Ne laser. The Raman signal was collected by a 100× objective (N.A. = 0.85) and detected by a E-cooled CCD sensor. The spectra were extracted with an integration time of 10 s for three times.

## 6. Micro-FTIR Measurement

The EOT spectra of Ag microhole arrays were acquired by an infrared microscopy system (Nicolet iNIO MX, Thermo Fisher Scientific, America) combined with Fourier-transform spectrometer possessing the wavenumber resolution of 8 $cm^{-1}$. The efficiency of micro-FTIR measurement was strengthened by a Cassegrain objective (15×, N.A. = 0.4). Meanwhile, an aperture (200 μm × 200 μm) was used to improve signal-to-noise ratio. The signal was obtained by a liquid $N_2$-cooled MCT photodetector.

## 7. Electrical Measurements

The electrical characteristics of the $MoS_2$ transistor were measured using a Lakeshore PS100 probe station and a Keysight B1500A semiconductor parameter analyzer. All of the data were measured at room-temperature.

# Figures in Supplementary Information

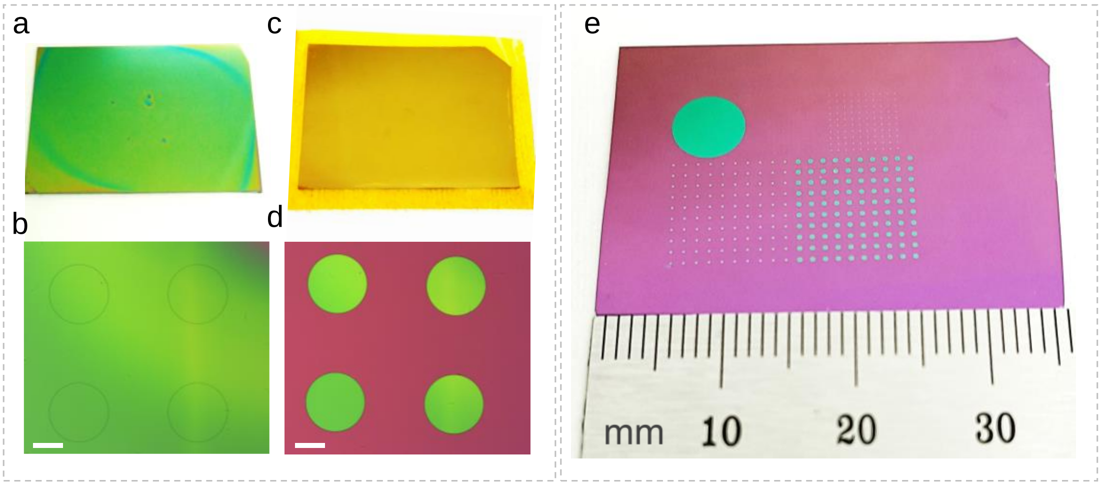


**Figure S1.** The photographs of resist nanokirigami based patterning process at different stages. (a) The sample after spin-coating PMMA on the HMDS-modified $SiO_2$/Si substrate. (b) The contour pattern array on PMMA layer after development with enlarged viewing. The size of single disk is 400 μm in diameter. (c) The patterned sample after PI tape pasting. (d) The corresponding fabricated PMMA microdisk array after selective peeling off. (e) The overview of fabricated sample after peeling off. Scale bars: (b, d) 200 μm.

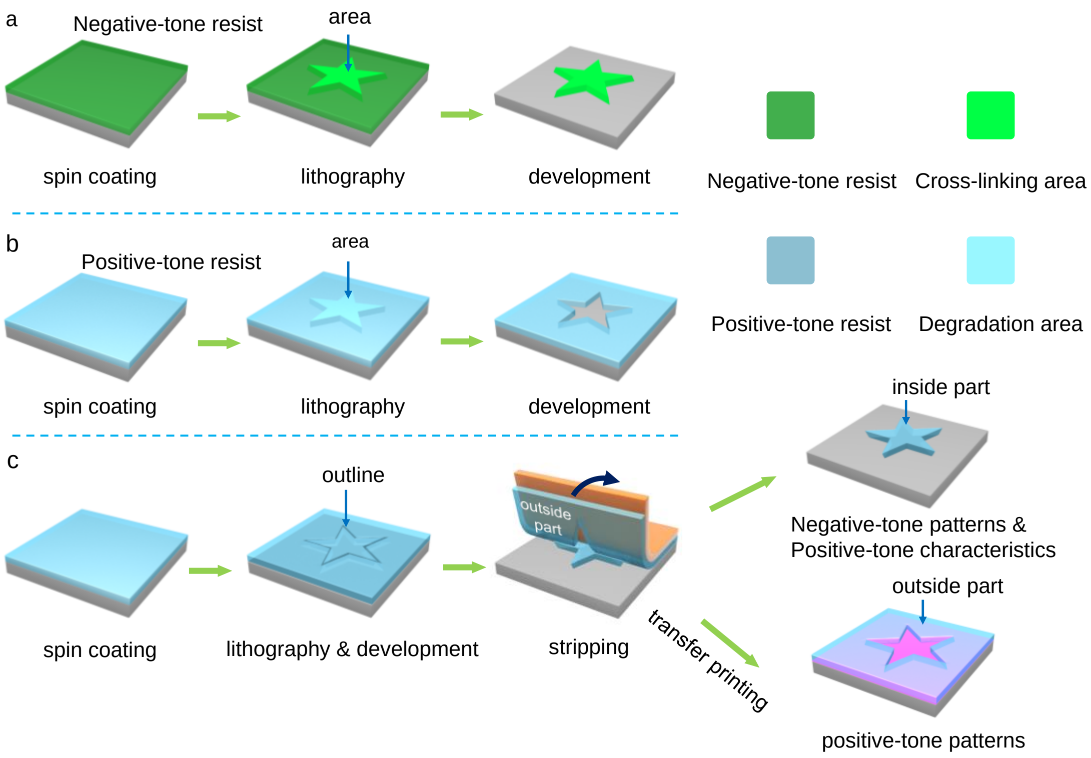


**Figure S2.** The comparison of resist nanokirigami with conventional positive-tone or negative-tone electron beam lithography. (a, b) Conventional electron beam lithography strategy for negative-tone or positive-tone resist, respectively. (c) Nanokirigami strategy for both positive-tone and negative-tone patterning.

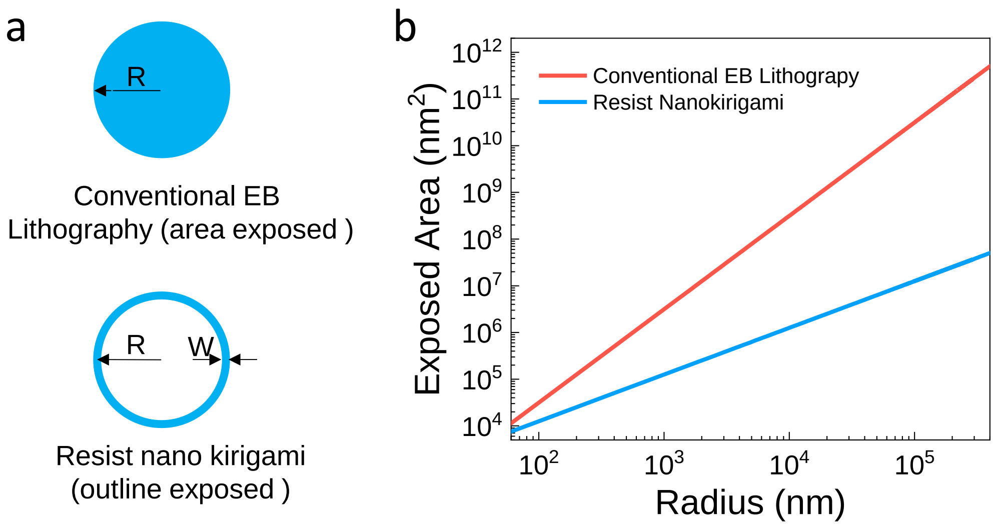


**Figure S3.** The exposure area of resist Nanokirigami is greatly reduced than that of traditional electron beam lithography. (a) the schematic of conventional electron beam lithography and ressit Nanokirigami, R is the radius of the structure, the width of the outline W is 20 nm. (b) To prepare a structure with a radius of R, traditional lithography and Resist NanoKirigami need to expose the area.

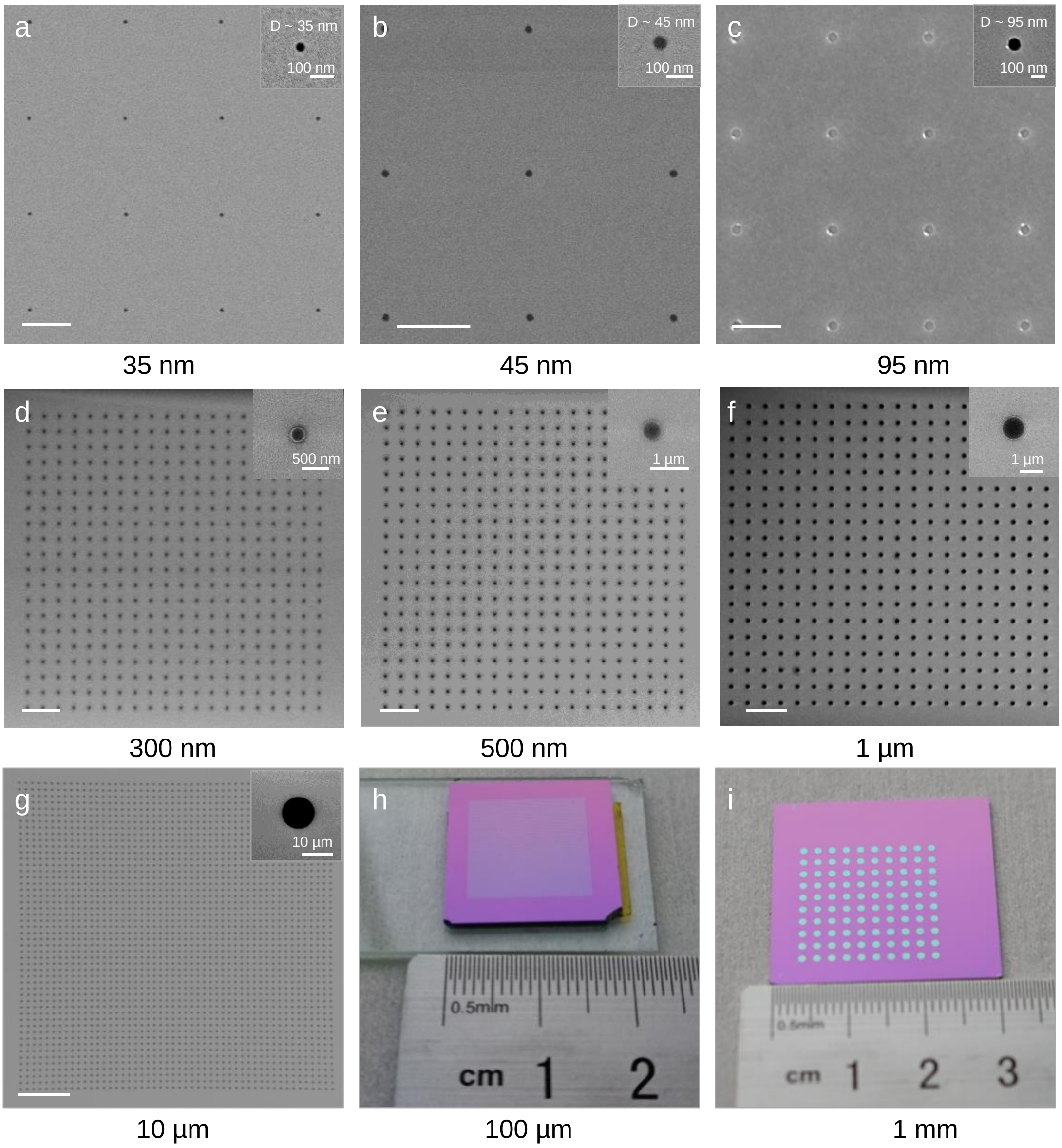


**Figure S4.** The SEM images and photographs of periodic PMMA disks varying size with the range from 35 nm to 1 mm. (a-c) Periodic Cr nanoholes with different sizes in diameter which were fabricated by resist nanokirigami based method we proposed. 35 nm in (a); 45 nm in (b); 95 nm in (c). Here, the obtained PMMA nanoposts at sub-100-nm scale would occur the transformation if they are directly irradiated by electron beam in SEM characterization, so we measured the diameter of nanoholes which are prepared by the pattern transfer of PMMA nanoposts based on the liftoff of 10-nm Cr. (d-g) The electron micrographs of fabricated PMMA disk arrays of which the sizes are 300 nm, 500 nm, 1 μm and 10 μm, respectively. The upper-right insets clearly show the corresponding enlarged micrographs of single structures in the array. (h, i) The photographs of periodic PMMA micro-disks with 100 μm and 1 mm in diameter. The photograph is captured by the digital camera of a smartphone. Scale bars: (a-c) 500 nm; (d, e) 5 μm; (f) 10 μm; (g) 200 μm.

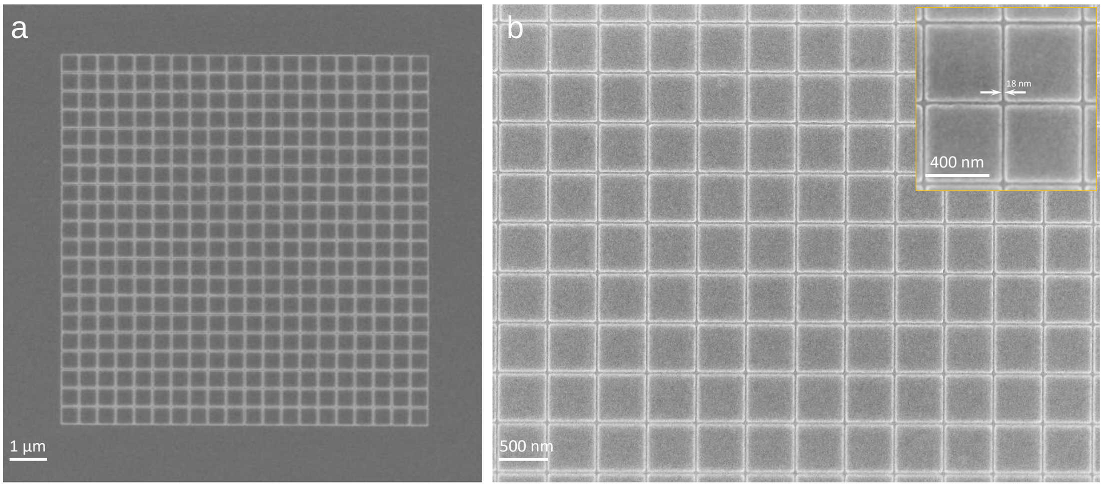


**Figure S5.** The SEM images the ultra-density nanostructures. (a) the ultra-density PMMA structures. (b) the magnitude SEM images in (a), the distance between the structures can down to 18 nm, that is hardly fabricated by conventional electron beam lithography. Noting, to meet the requirements of SEM characterization for sample conductivity, 8 nm of Cr was evaporated on the surface of the sample.

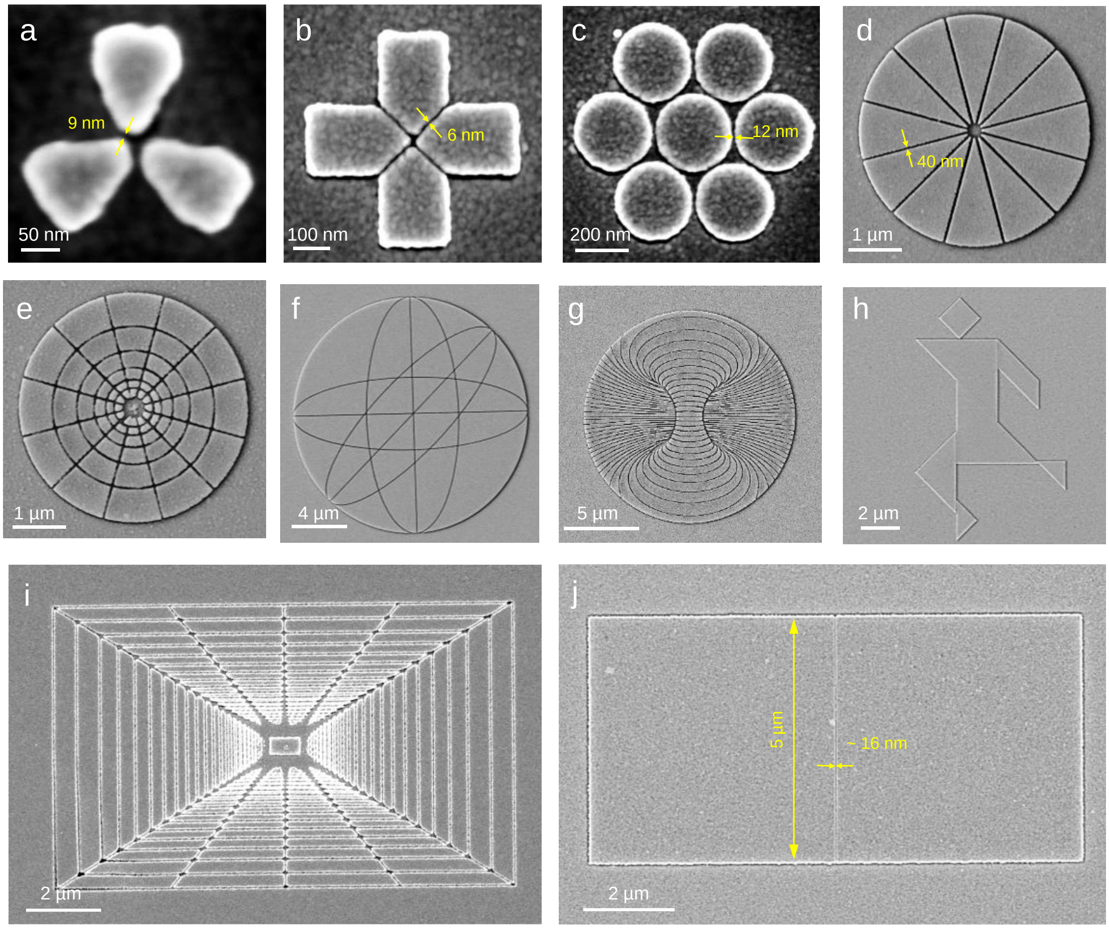


**Figure S6.** The gallery of multiscale metallic assemblies fabricated by the direct deposition on defined PMMA post templates. (a-c) Single plasmonic trimer, tetramer and heptamer with ultrasmall nanogaps which are difficult to achieve with traditional EBL processes. (d-j) The other complex multiscale metallic assemblies with nanogaps on PMMA post templates prepared by our method. In gap parts, the share-boundary rule was used in layout designs.

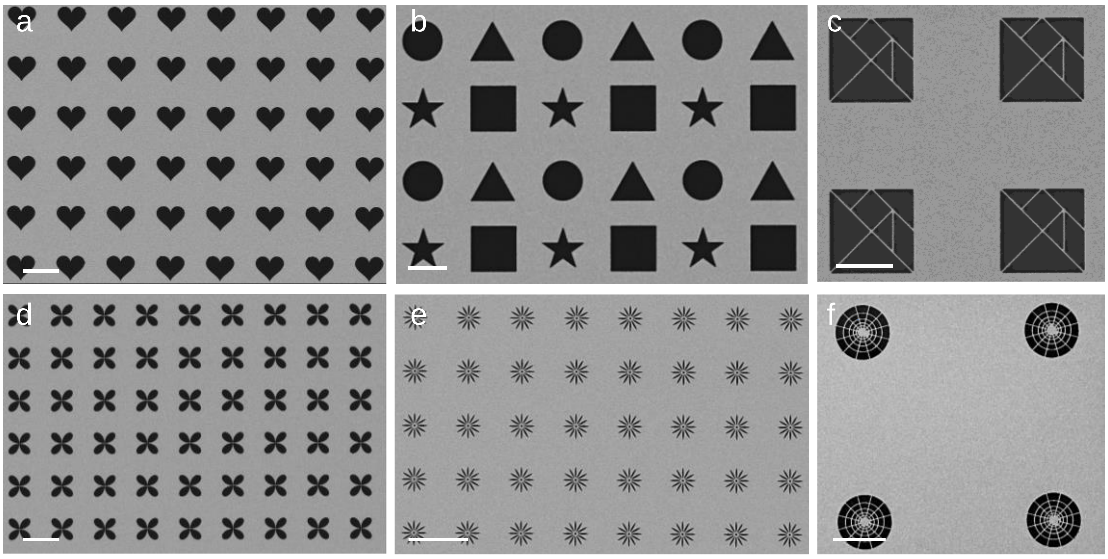


**Figure S7.** Multiscale complex inverse structure arrays. (a) Heart-shaped microhole array; (b) polygon-suit microhole array. The suit contains circle, triangle, box and star. (c-f) The assembled inverse structure array. (c) Jigsaw puzzle; (d) four-leaf shape; (e) sun shape; (f) spider web. In gap parts, the share-boundary rule was used in layout designs. Scale bars: (a, d, e) 10 μm; (b, c) 4 μm; (f) 2 μm.

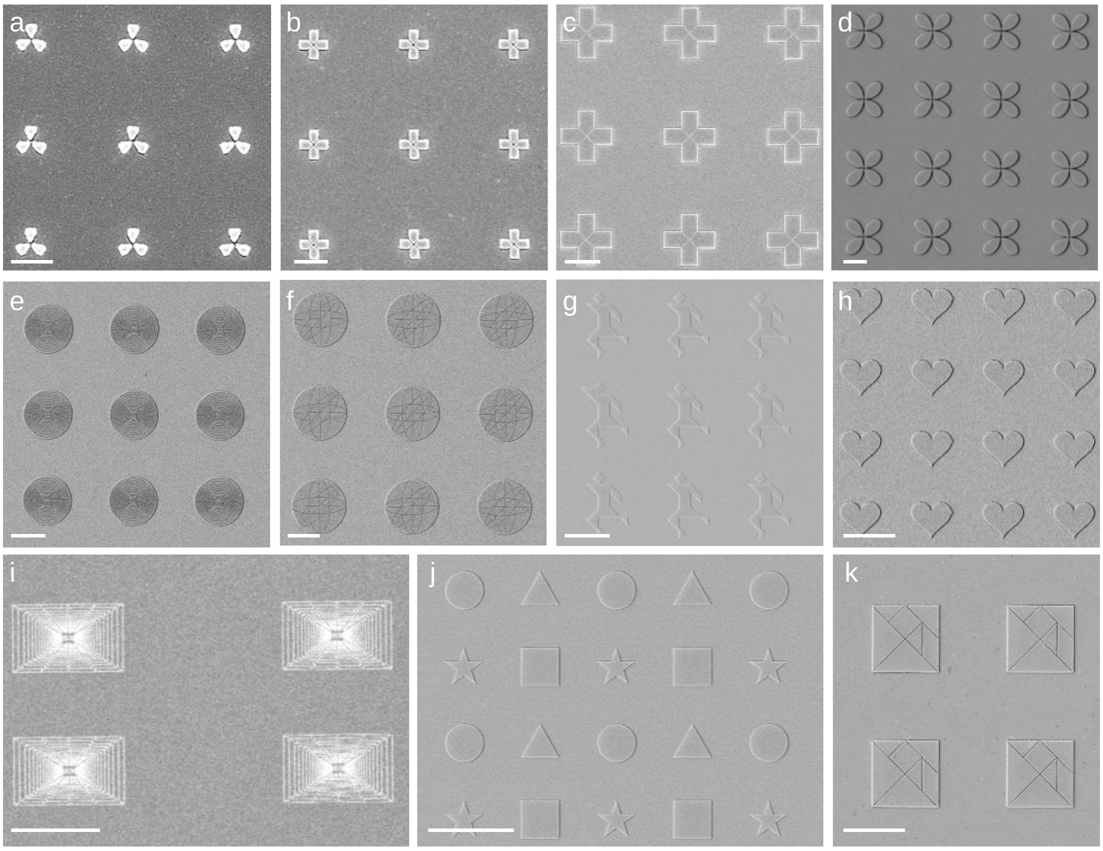


**Figure S8.** Multiscale complex structures arrays fabricated by the direct metal deposition on resist nanokirigami defined PMMA post templates. (a-d) Typical trimer (a), tetramer (b-d) and more complex pattern (d-g, i, k) arrays. All of these structures are multiscale because they are the assemblies of nanoscale and microscale tiles with nanogaps. (h, j) The periodic heart-shape and polygon microscale

structures. This kind of structures are multiscale as well because of some nanoscale features (*e.g.*, convex and concave nanoscale corners) in single structure. Scale bars: (a) 400 nm; (b) 500 nm; (c, d) 2μm; (e-k) 10 μm.

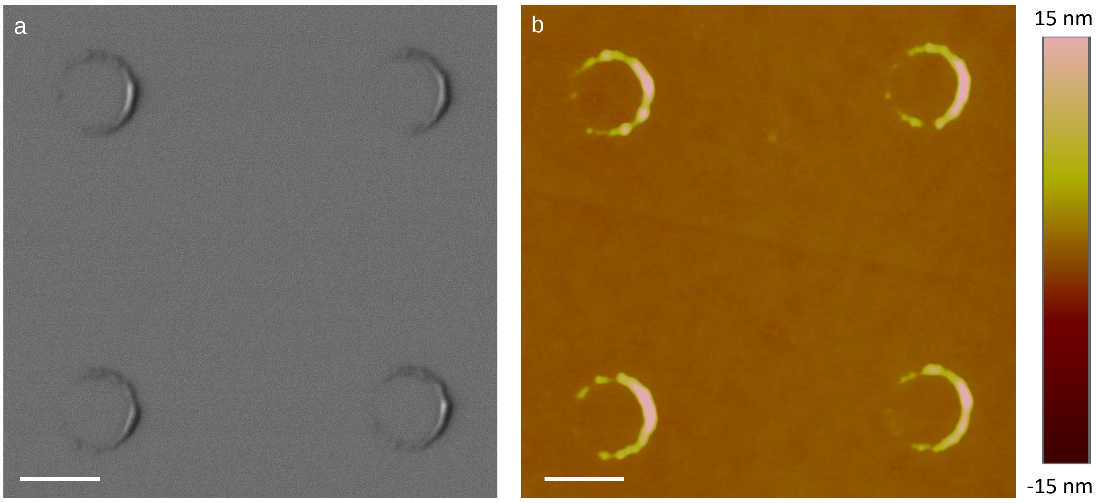


**Figure S9.** Evidence of adhesive transition caused by electron-beam radiation area in control experiment. (a) The ring-like PMMA residues patterns after peeled off the top PMMA layer. The dark area is high adhesion caused by the irradiation of focused electron beam in lithography. Due to the short of electron dose and has not developer with chemical development, inside and outside PMMA were completely peeled off from substrate. (b) The corresponding AFM image of the height sense. All scalebar: 500 nm.

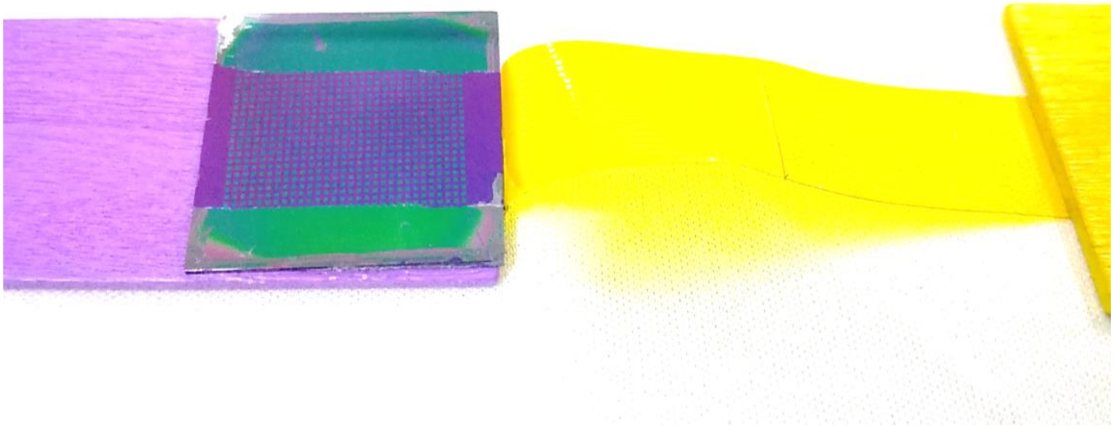

**Figure S10.** The photograph of patterned sample after peeling test.

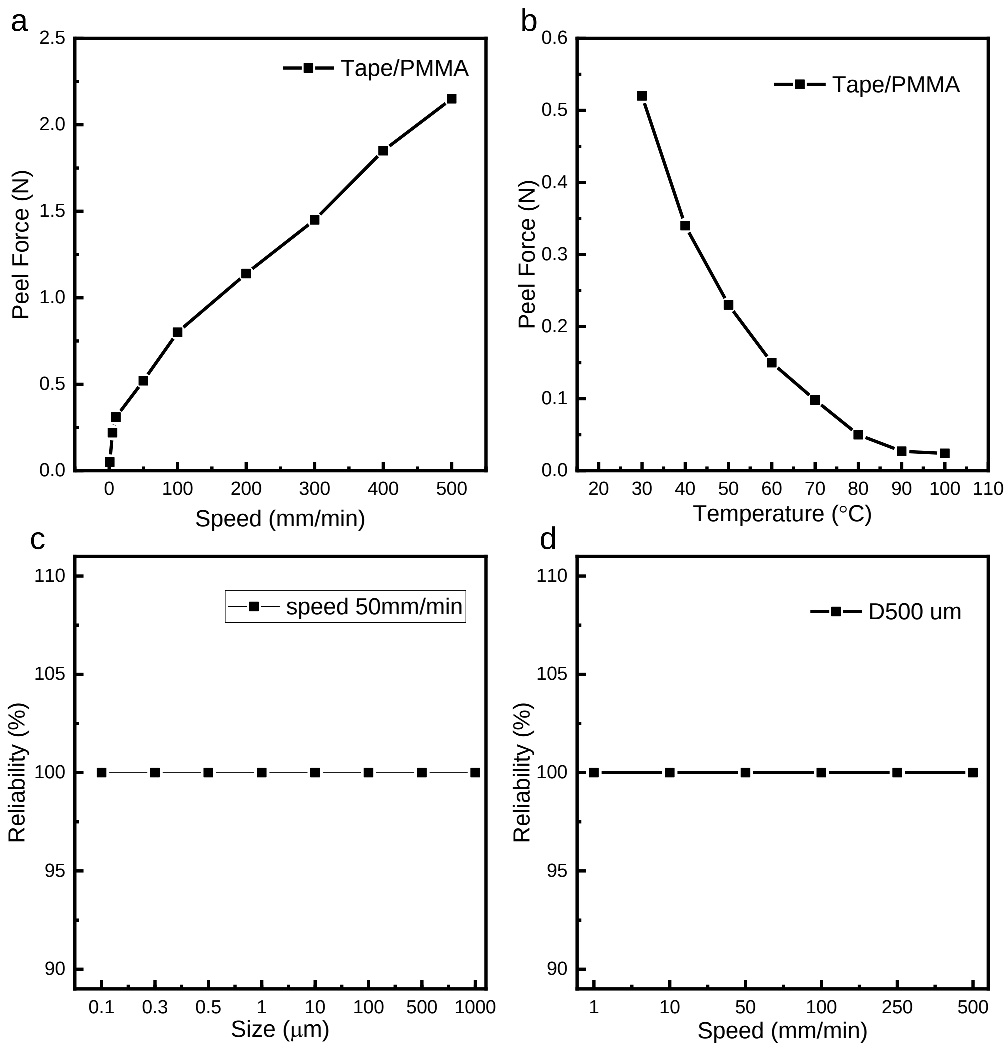


**Figure S11.** Adhesion analysis of thermal release tape and the reliability statistic of resist nanokirigami process using this tape. (a) The adhesion force of the thermal release tape pasted on PMMA with different peeling rates. (b) The adhesion force function of thermal release tape pasted on PMMA with respect to working temperature. (c) The reliability of our method to fabricate PMMA disk with varied-size disk in diameter from 100 nm to 1 mm. (d) The yield of 500-nm disks in statistic arrays at different rates during peeling off.

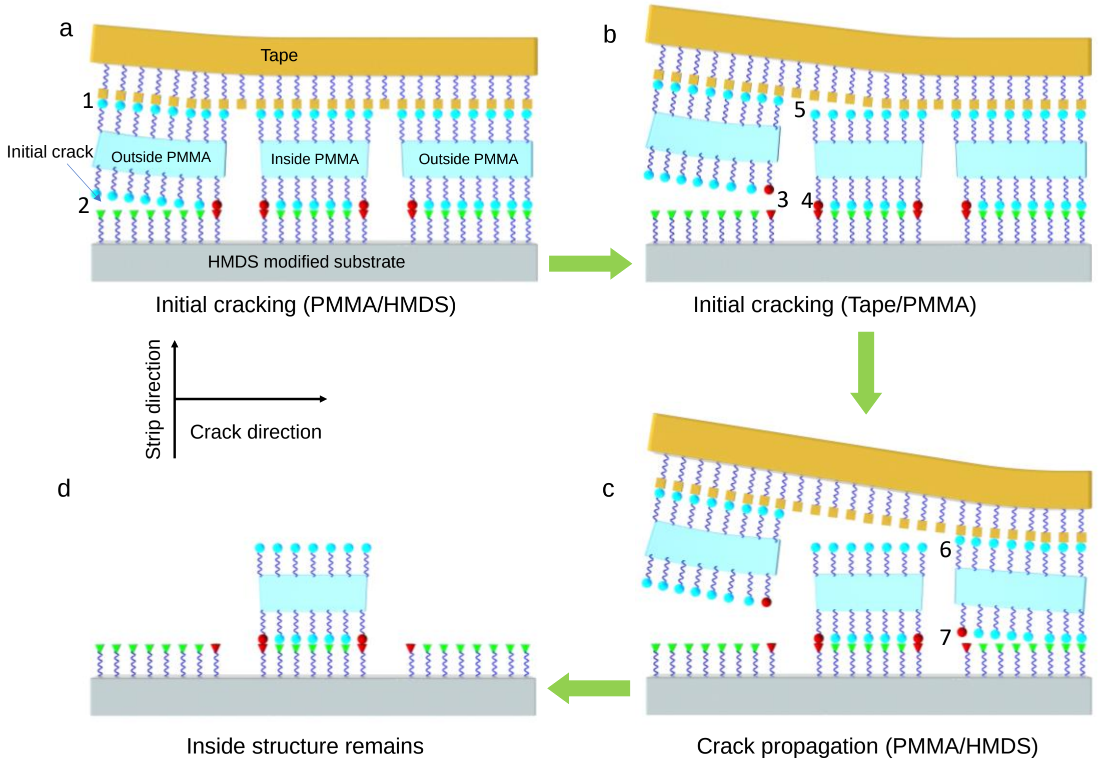


**Figure S12.** The crack propagation at the PMMA/HMDS and Tape/PMMA interface. (a) At the beginning of peeling off using PI tape, the existence of defects at the edge of sample and low adhesion (green) at the PMMA/substrate interface promote to the initiation of crack at PMMA/substrate interface (No.2 site). (b) As the crack front propagates to No.3 site, although the PMMA/substrate at this site transits to be high adhesion (red) due to focused electron-beam irradiation, PMMA is still peeled off because of the stress concentration at the front of crack in case of the appearance of the convergent geometry orienting to No.3 site and the opening expansion during peeling off. In contrary, the condition of crack propagation is not available at No. 4 site, so the crack cannot propagate at the interface between inside PMMA structure and substrate. Meanwhile, for PMMA/tape interface, the rounded corner at top edge (No.5 site) of inside PMMA structure served as a convergent notch, and opening of the notch will be expand when the stripping continues. Therefore, a new crack is formed at the PMMA/tape interface. (c) As the crack propagation continues, the tape completely detaches from the inside PMMA structure. Attributed to the continuousness of outside PMMA film, the crack at the outside PMMA/substrate interface still propagates, so No.7 site meet the condition of crack propagation and No.6 site presents high adhesion. (d) Finally, the outside PMMA film is peeled off and the inside PMMA structure remains on the substrate.

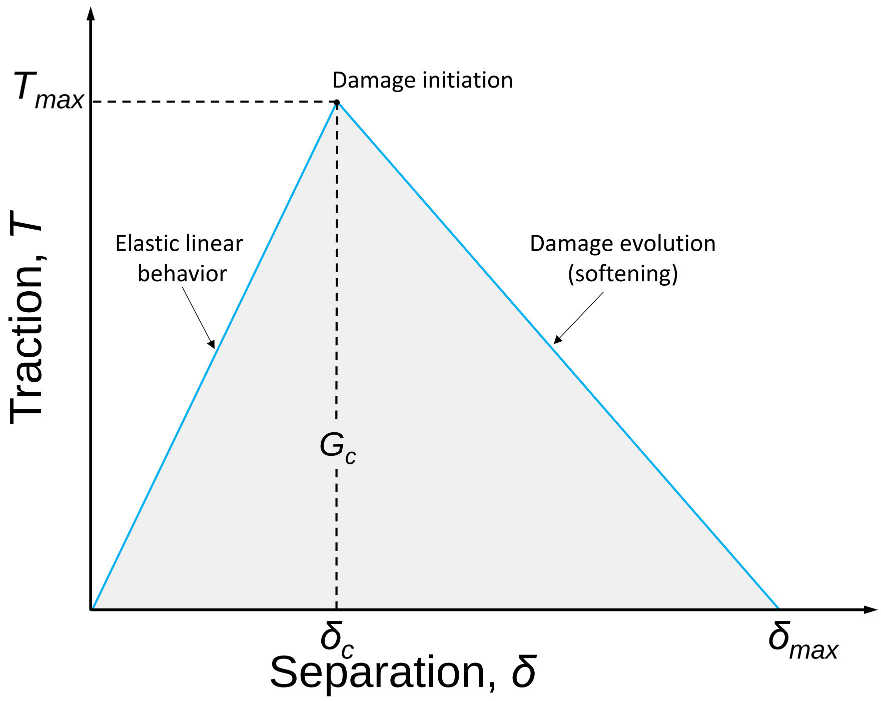


**Figure S13.** Traction-Separation (T-δ) law of cohesive zone model. The constitutive behavior of the interface is assumed to be a bilinear traction-separation (*T-δ*) law consisting of an elastic phase and a softening phase. The interface traction force *T* as a function of the separation vector *δ*. The curve of traction-separation shows the elastic transformation in case of $T<T_{max}$. When $T \geq T_{max}$, the interfacial damage initiates and continues to expand with a softening manner. $G_c$ is the adhesion work (the filled area under curve marked by grey colour). The width of the tape is 10 mm.

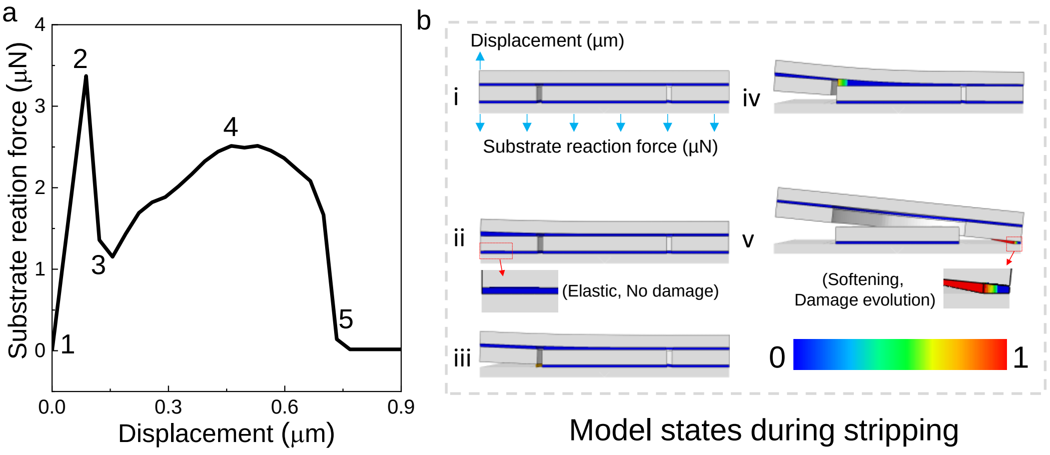


**Figure S14.** The analysis of interfacial damage in FEM simulation. (a) The diagram of reaction force on substrate as a function of displacement. As shown in the diagram, there are five key scenarios in the peeling process. P1 represents the start of peeling; P2 presents the initiation of facture at the interface between PMMA and substrate; P3 means the complete delamination of outside PMMA film on the side of start peeling; P4 is the damage generation at PI/PMMA interface when the fracture front propagates to the inside structure; P5 shows the complete peeling off of outside PMMA film. (b) The interfacial damage distribution near the stages of P1 (b-i), P2 (b-ii), P3 (b-iii), P4 (b-iv) and P5 (b-v). Colour bar represents the normalized level of interface damages, 0 means no damage, 1 means complete separation.

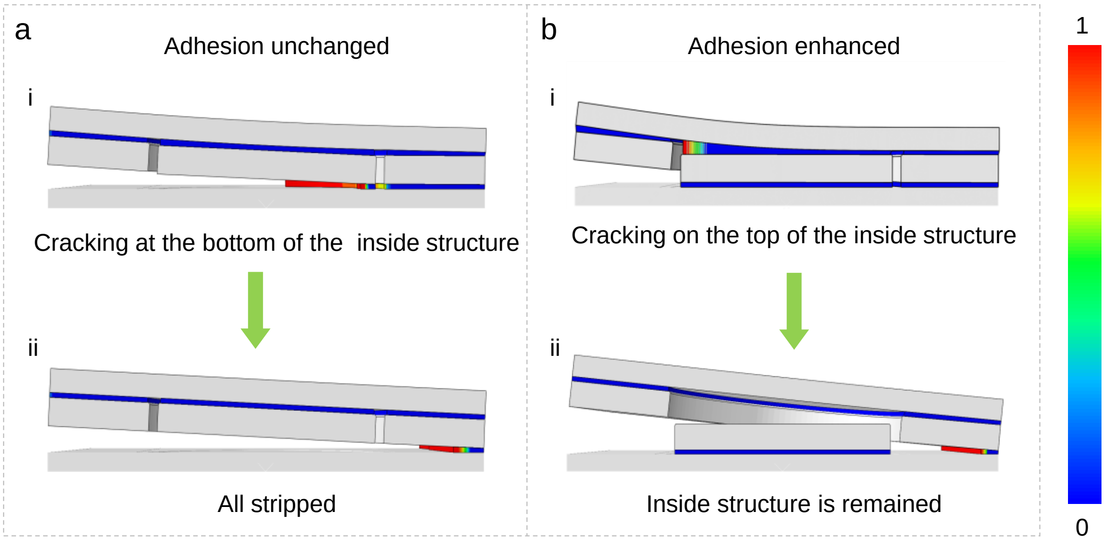


**Figure S15.** Whether the adhesion of the irradiated area is enhanced or not has the effect on the simulation results (a) the simulation model just has the characteristic of a round profile and the adhesion at the PMMA/substrate interface of the irradiated area not enhanced. (b) The model has the characteristics of a round profile and local adhesion enhanced.

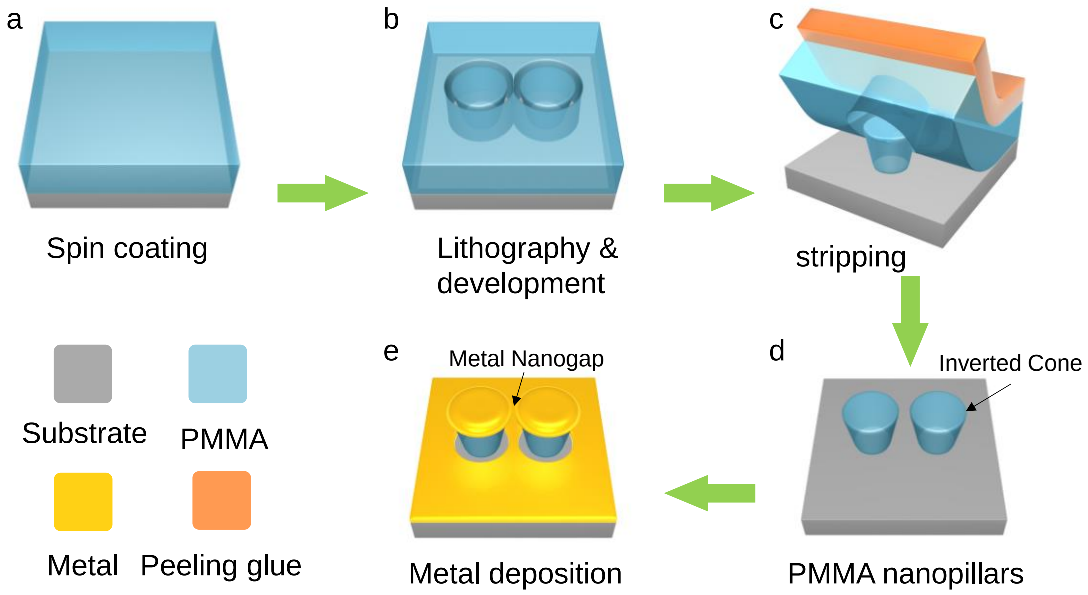


**Figure S16.** 3D models to schematically show the process of defining freestanding metallic nanogaps with resist nanokirigami based method we proposed. The key advantages of our method to fabricate ultrasmall plasmonic nanogaps are stated in main text. (1) The undercut profile of defined PMMA nanoposts with the use of bilayer PMMA resist in patterning. (2) The contour exposure greatly mitigates the proximity effect. (3) The resultant gap size in metal dimers can be further shrunk after metal deposition due to the lateral growth during metal evaporation.

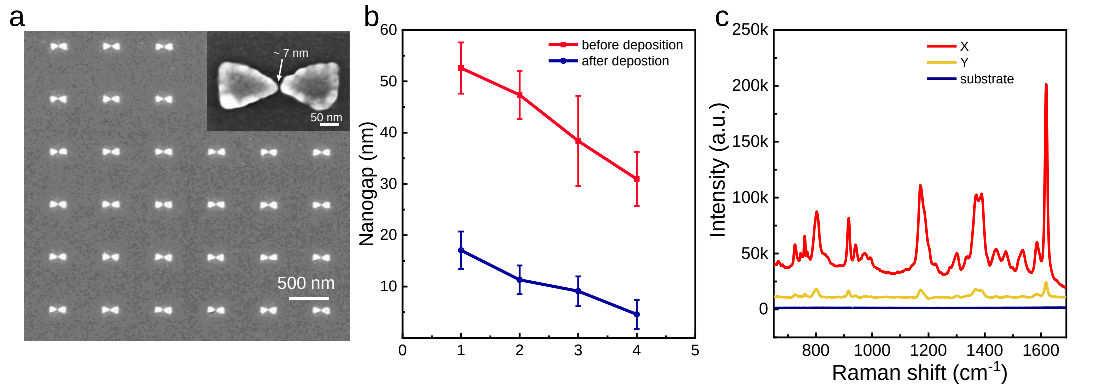


**Figure S17.** The SERS performance of fabricated plasmonic nanobowtie with sub-10-nm gap. (a) SEM image of arrayed nanobowties with sub-10-nm gap. The inset clearly shows the gap size of ~ 7-nm in single nanobowtie. (b) Plot of statistic gap sizes in PMMA post template before and after 30-nm Au deposition. (c) Polarized SERS measurements of Au nanobowtie with 7-nm gap.

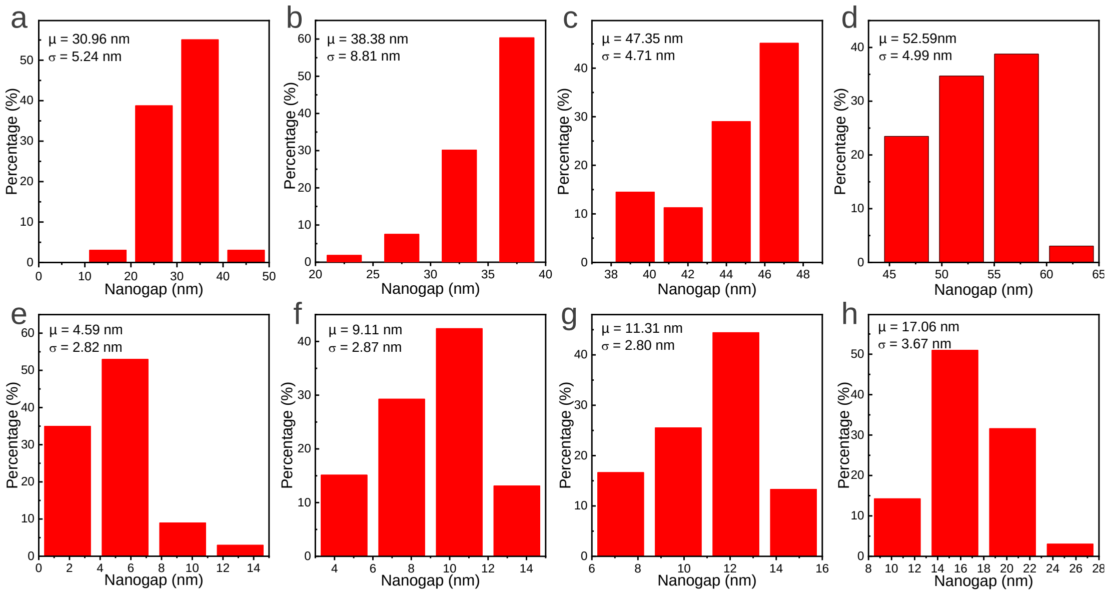


**Figure S18.** Statistics of gap sizes in Au nanobowtie before and after Au deposition. The statistic results of gap size in the as-fabricated PMMA nanopost dimers before (a-d) and after (e-f) 30-nm Au deposition. Each histogram is extracted from the measured data of 80 structures (sampling volume).

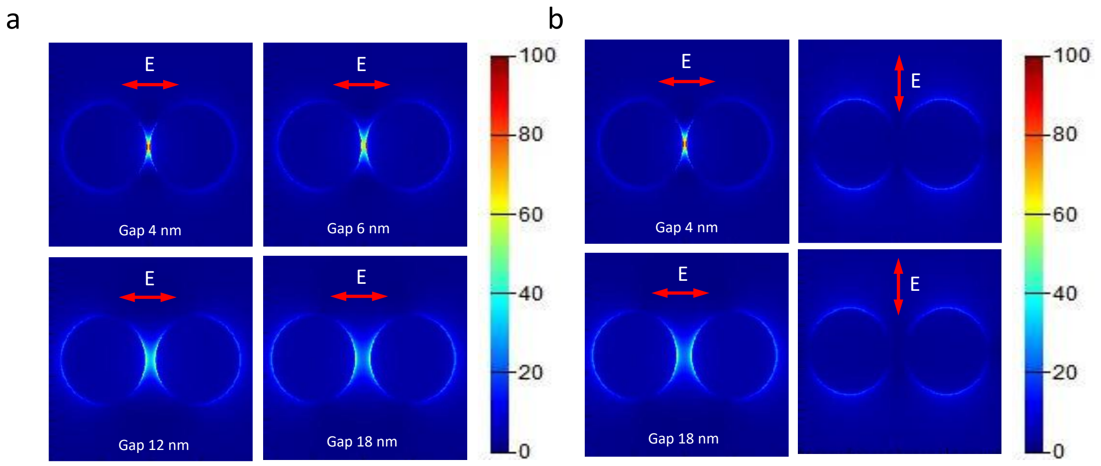


**Figure S19.** Electric field distribution of Au nanodisk dimer with freestanding nanogaps varying size. (a) The enhancement factor ($E/E_0$) of electric field in the hotspot of varied gap size in Au nanodisk dimer at 633 nm. (b) $E/E_0$ factor of 4-nm and 18-nm gaps in nanodisk dimers with the linear excitation of X and Y polarization at 633 nm.

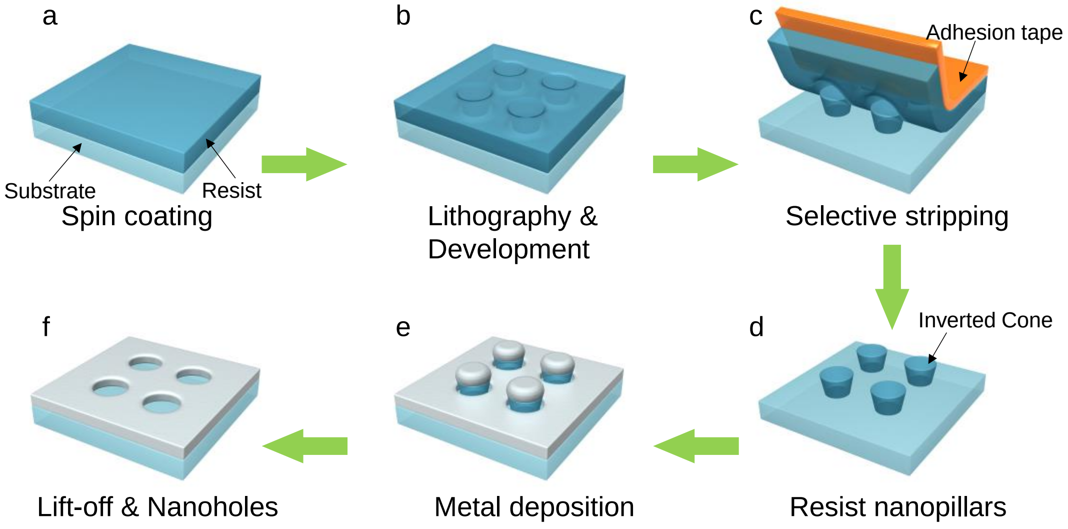


**Figure S20.** Schematic flow-charts to show the strategy of fabricating metallic inverse structures using our method. Compared to the negative-tone-resist-based liftoff to fabricate inverse structures, our method has some advantages as follow: (1) The resultant positive-tone resist (*e.g.*, PMMA) post have the undercut profile to greatly mitigate the sidewall deposition due to the enabling of using bilayer resist that is difficult to achieve in negative-tone resists. (2) The positive-tone resist posts are facile to be dissolved during liftoff, but it is difficult in negative-tone resists because of the change from soluble to insoluble after exposure.

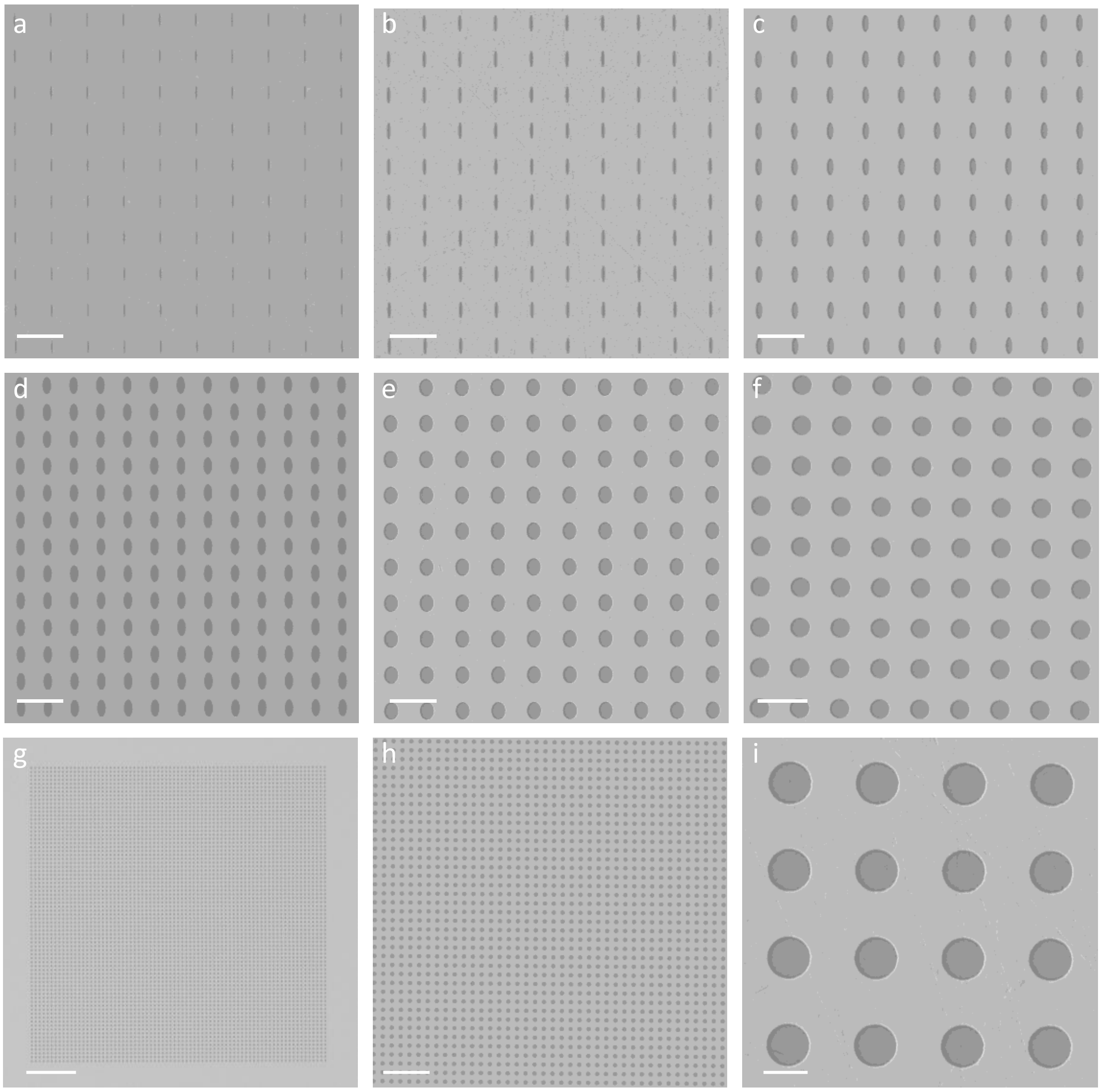


**Figure S21.** Large-area Ag elliptical microhole arrays with varied X-axes length in ellipsoid. (a-f) SEM images of elliptical microhole arrays with varying ovalities. All ellipsoids have the fixed length in Y-axes, and the lengths in X-axes are 200 nm (a), 400 nm (b), 800 nm (c), 1000 nm (d), 1600 nm (e) and 2000 nm (f), respectively. (g-i) The electron micrographs of periodic microholes with the diameter of 2 μm with different magnifications. The pitch is 4 μm. Scale bars: (a-f) 5 μm; (g) 50 μm; (h) 20 μm; (i) 2 μm.

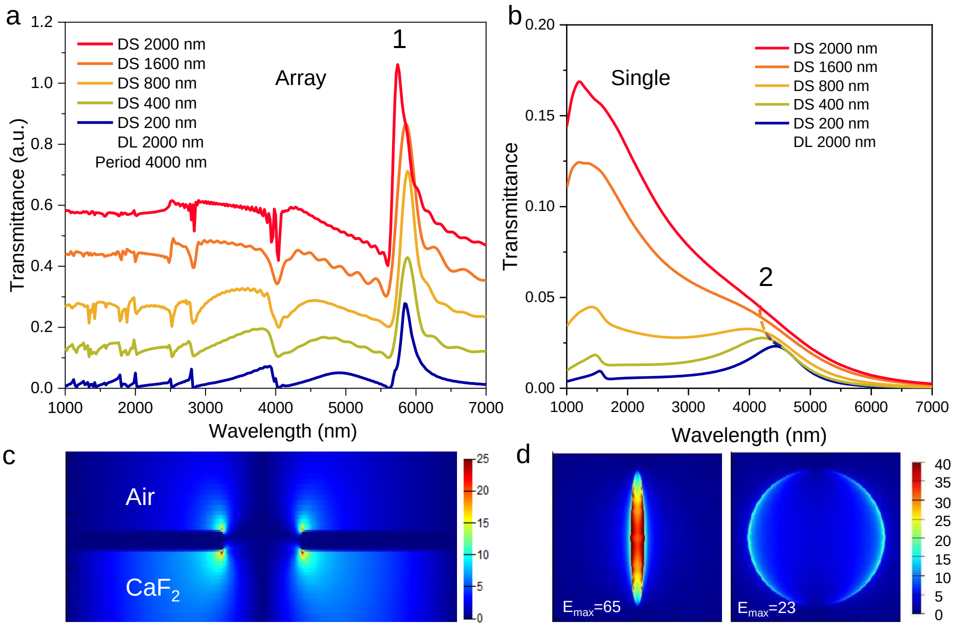


**Figure S22.** The simulated transmittance of arrayed Ag elliptical holes and single one. (a) The transmission spectra of periodic elliptical microholes varying the ovalities. The length in Y axes is constant of 2 μm, and the lengths in X-axes are 200, 400, 800, 1600 and 2000 nm, respectively. (b) The transmittance of serial single microholes of which the sizes are corresponded to those in diagram (a). DS, DL represent the length in X-axis and the length in Y-axes, respectively. (c) $E_z$ mapping at Peak 1 from side viewing normal to XZ plane. The $E_z$ profile means the resonance of propagating surface plasmon (SPP) at the Ag/$CaF_2$ interface. With the further analysis of resonance order reported in the previous literature[3], the order of SPP is the $(1, 0)_{CaF2}$ mode along the long axes of ellipsoid. (d) The calculated $E/E_0$ topography of single Ag microhole on film. The left panel is ellipsoid with DS = 200 nm and DL = 2000 nm, and the right panel is the circle with the diameter of 2000 nm.

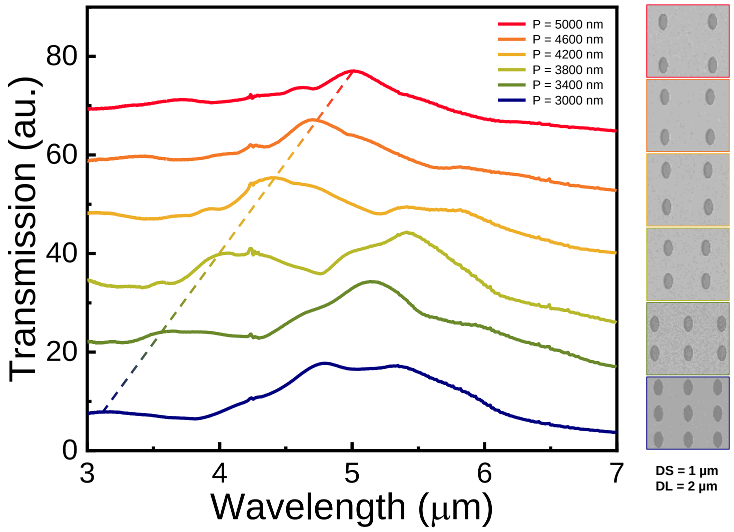


**Figure S23.** Spectral response of EOT devices based on Ag elliptical microholes with different pitches. The redshift and peak positions match the geometric parameter of pitch at Y axes, which means that the mark peak is (1, 0) lattice mode at the Ag/air interface. DS and DL represent the length in X-axis and the length in Y-axes, respectively.

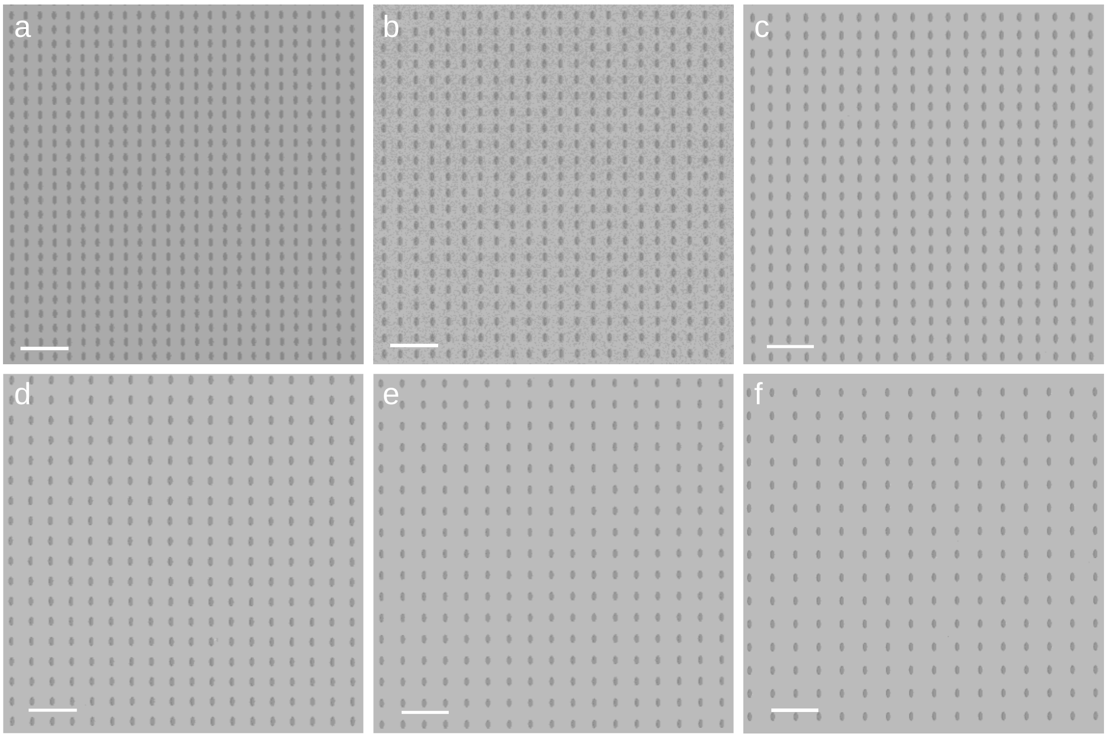


**Figure S24.** The electron micrographs of large-area Ag elliptical microhole arrays with different pitches in the EOT device. (a-f) The pitch in array increases from 3 μm to 5 μm with the increment of 400 nm. 3 μm in (a), 3.4 μm in (b), 3.8 μm in (c), 4.2 μm in (d), 4.6 μm in (e), 5 μm in (f). Scale bar: 10 μm.

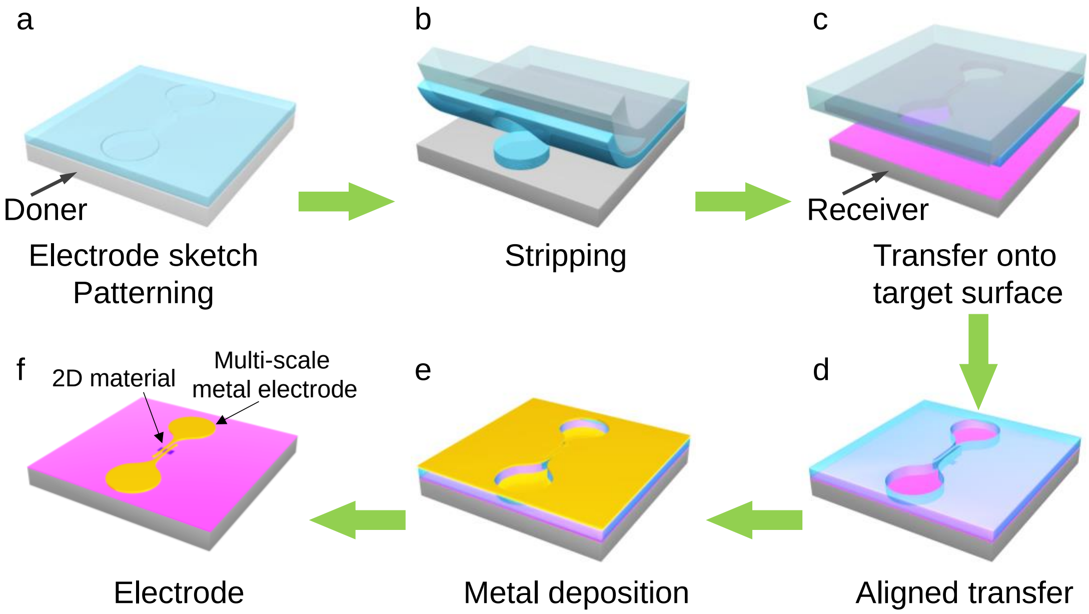


**Figure S25.** Schematic showing the fabrication of $MoS_2$ transistor. (a) Contour patterning of electrode on the doner substrate. (b) Peeling off outside PMMA film to prepare stencil template. (c) Transferring the PMMA stencil onto $MoS_2$ flake with alignment. (d) Printing the stencil onto $MoS_2$ on the acceptor substrate after retrieving PDMS. The two final steps are finishing Au deposition (e) and liftoff (f), respectively.

## References

1. Nicholas T, Boyajian D, Chen SE *et al.* Finite element modeling of mode I failure of the single contoured cantilever CFRP-reinforced concrete beam. *J Struct* 2013; **2013**: 1-8.

2. Torres JM, Stafford CM and Vogt BD. Elastic modulus of amorphous polymer thin films：relationship to the glass transition temperature. *Acs Nano* 2009; **3**: 2677–85.

3. Gordon R, Brolo AG, Sinton D *et al.* Resonant optical transmission through hole-arrays in metal films: physics and applications. *Laser Photonics Rev* 2010; **4**: 311-35.
